# Reducing the structure bias of RNA-Seq reveals a large number of non-annotated non-coding RNA

**DOI:** 10.1093/nar/gkaa028

**Published:** 2020-01-25

**Authors:** Vincent Boivin, Gaspard Reulet, Olivier Boisvert, Sonia Couture, Sherif Abou Elela, Michelle S Scott

**Affiliations:** 1 Département de biochimie et génomique fonctionnelle, Faculté de médecine et des sciences de la santé, Université de Sherbrooke, Sherbrooke, QC J1E 4K8, Canada; 2 Département de microbiologie et d’infectiologie, Faculté de médecine et des sciences de la santé, Université de Sherbrooke, Sherbrooke, QC J1E 4K8, Canada

## Abstract

The study of RNA expression is the fastest growing area of genomic research. However, despite the dramatic increase in the number of sequenced transcriptomes, we still do not have accurate estimates of the number and expression levels of non-coding RNA genes. Non-coding transcripts are often overlooked due to incomplete genome annotation. In this study, we use annotation-independent detection of RNA reads generated using a reverse transcriptase with low structure bias to identify non-coding RNA. Transcripts between 20 and 500 nucleotides were filtered and crosschecked with non-coding RNA annotations revealing 111 non-annotated non-coding RNAs expressed in different cell lines and tissues. Inspecting the sequence and structural features of these transcripts indicated that 60% of these transcripts correspond to new snoRNA and tRNA-like genes. The identified genes exhibited features of their respective families in terms of structure, expression, conservation and response to depletion of interacting proteins. Together, our data reveal a new group of RNA that are difficult to detect using standard gene prediction and RNA sequencing techniques, suggesting that reliance on actual gene annotation and sequencing techniques distorts the perceived architecture of the human transcriptome.

## INTRODUCTION

Gene annotation is the blueprint of the human genome upon which gene expression analyses are performed (1,2). Translation open reading frames were the primary scaffold for annotating protein-coding genes that were later validated through transcriptomic and genetic analysis (3–5). As a result, the overall number of protein coding genes in the human genome now stands at ∼20 000 with little change in their annotation in the past ten years (3,6). In contrast, the annotation of the wide spectrum of non-translated genes has proven much more challenging. In general, annotation of non-coding RNAs (ncRNAs) depends either on *in silico* prediction programs that scan for known features of ncRNA, or evidence of expression. Standard RNA sequencing pipelines use general gene annotation sets from databases like GENCODE (4) and RefSeq (5) to assign sequencing reads to specific genes, enabling the estimation of their expression. As a result, these pipelines discard aligned reads that map to non-annotated genes. For this reason, having a complete annotation set is essential to correctly evaluate the transcriptomic landscape with RNA-Seq data.

Some specialized databases such as RNAcentral aggregate annotation information from a multitude of databases, in order to produce a more inclusive depiction of non-coding RNA annotations (7). Using this type of annotation set increases the number of reads assigned to ncRNA but might also incorporate errors generated by unreliable prediction methods. Even when the prediction of ncRNA is possible, many non-coding RNAs exist in multiple copies, making it difficult to distinguish the active copies responsible for the non-coding transcripts and potential pseudogenes (8). Furthermore, the ncRNA are often hidden in the introns, and sometimes exons, of protein coding genes and their rapidly evolving nature makes them difficult to confirm based on interspecies conservation (9–11). Ultimately, a genomic locus should be annotated as a gene if it produces RNA that accumulates as a stable independent entity that is reproducibly detectable. However, the highly structured nature of non-coding RNA makes their detection unreliable with standard sequencing techniques.

Current sequencing techniques use retroviral reverse transcriptases that often stall when they encounter RNA structures or modified nucleotides and are prone to ligation bias (12,13). As a result, standard sequencing techniques like Illumina TruSeq are biased towards the detection of less structured RNA such as protein-coding transcripts, and do not always provide a representative read distribution across the full length of non-coding RNA (14). This makes *de novo* prediction of ncRNA based on transcript definition very difficult and increases the reliance on a predetermined definition of these RNA. Using highly processive and structure-tolerant reverse transcriptases like the thermostable group II intron reverse transcriptase (TGIRT) permits the sequencing of full-length noncoding RNAs (14,15). This method of sequencing (TGIRT-Seq) does not require ligation of adapters to the RNA but instead uses the proficient template switching activity of TGIRT to couple adapter addition to the 3′ terminal nucleotide of an RNA template. As a result, this method dramatically increases the number of detected non-coding RNA, especially snoRNA and tRNA. Indeed, direct comparison of the same RNA sequenced by either TGIRT-Seq or TruSeq techniques indicates that the number of reads assigned to snoRNA and tRNA increases 4–17 times using TGIRT-Seq (16). Most importantly, TGIRT-Seq faithfully reproduced the RNA ranks predicted using biochemical methods and gene specific studies (16).

In this study, we take advantage of the homogeneous full-length distribution of the sequencing reads produced by TGIRT-Seq to carry out *de novo* identification of RNA transcripts independent of pre-established annotation. To accurately identify the natural termini of ncRNAs, increase confidence in transcript definition and have a greater sequencing depth of ncRNAs, sequencing was performed using non-fragmented RNA, derived from the ovarian cancer cell line, SKOV3ip1 (16,17). Overall, we identified a total of 1212 high-confidence distinct transcripts, many of which mapped to multiple loci. Importantly, 111 of the newly identified transcripts are produced from distinct previously non-annotated small non-coding RNA genes (sncRNA) we termed non-annotated RNAs or NA_RNAs. Sequence and structural similarity analyses indicate that 60% of these 111 NA_RNAs correspond to previously unidentified snoRNA or tRNA-like genes. The rest of the new transcripts correspond to transcribed ribosomal RNA (rRNA) spacers or display no homology in sequence or structure with any annotated genes. In general, these non-annotated tRNA (NA_tRNA) genes are well conserved in vertebrates while non-annotated snoRNA (NA_snoRNA) are mostly primate-specific. In addition, the NA_H/ACA snoRNAs are affected by the depletion of Dyskerin, a core component of H/ACA snoRNPs, confirming their integration into functional H/ACA snoRNP complexes. Comparing the structure of the newly identified transcripts to known tRNAs and snoRNAs revealed some structural or sequence anomalies explaining why the new transcripts were overlooked by standard function predictors and suggesting possible non-canonical functions of these RNAs. Together, the results indicate that the catalogue of human non-coding RNA is far from complete and underscore the power of *de novo* transcript detection in specialized sequencing techniques as a tool for gene definition.

## MATERIALS AND METHODS

### RNA-Seq dataset accessions

The SKOV3ip1 and INOF TGIRT RNA-Seq datasets were obtained from NCBI Gene Expression Omnibus (GEO) series GSE99065 (16) including datasets GSM2631743 and GSM2631744 for the SKOV3ip1 non-fragmented ribodepleted TGIRT-Seq datasets, GSM2631741 and GSM2631742 for the SKOV3ip1 fragmented ribodepleted TGIRT-Seq datasets, GSM2631745 and GSM2631746 for the SKOV3ip1 fragmented ribodepleted classical RNA-Seq and GSM2997959 for the INOF fragmented ribodepleted TGIRT-Seq datasets. The human reference RNA and brain RNA ribodepleted fragmented TGIRT-Seq datasets were obtained from the NCBI Short Read Archive (SRA) (SRR2912443, SRR2912444 and SRR2912446 for the human reference RNA datasets and SRR2912479, SRR2912481 and SRR2912483 for the brain RNA datasets) (14) Hydro-tRNAseq datasets are available from GEO (sample accessions GSM2521595, GSM2521596, GSM2521597 and GSM2521598) (18). Datasets for the YAMAT-Seq were obtained from SRA (SRR5168440, SRR5168441 and SRR5168442) (19) and the demethylase TGIRT-Seq dataset from GEO (GSM1624818 and GSM1624819) (20). All RNA-seq datasets considered in this study are listed in Supplementary Table S1.

### RNA-Seq data processing

All datasets were analyzed using the same computational pipeline. Fastq files were checked for quality using FastQC and trimmed using Cutadapt (21) and Trimmomatic (22) to remove Illumina sequencing adapters and portions of reads of low quality, respectively. Reads were aligned using STAR (23) and reads not aligned were aligned once again with Bowtie2 (24) as described in (16). All the mapped reads were then merged using Samtools merge (25). The reference genome used was hg38 and the reference annotation to build the STAR alignment index was taken from Ensembl (v87) (6). Parameters values for all tools used are given in Supplementary Table S2.

### Read cluster detection and selection

The Blockbuster tool was used for read cluster detection (26). To prepare the Blockbuster input bed files, the SKOV3ip1 non-fragmented ribodepleted TGIRT-Seq alignment files were converted to bed format using the bamtobed tool from the bedtools suite (27) followed by an in-house script which outputs the strand-specific read-pair coverage per genomic region. Read clusters were identified using Blockbuster (26) on the bed file with parameters: -format 1 -print 1 -minBlockHeight 100 -tagFilter 50. The read cluster detection pipeline was implemented with the workflow management system Snakemake (28) and is available at https://github.com/GaspardR/snakemake_blockbuster.

### Gene expression quantification

To optimize the expression quantification of small and mid-size non-coding RNAs, gene expression estimation in TPM was produced with the CountCorrector pipeline CoCo (v 0.2.1p4) (parameters: -c both –strand 1 –paired) which corrects read assignment for embedded and multimapped genes (29). The annotation provided to CoCo was the Ensembl (v87) annotation bonified with the 111 new sncRNA gene entries as well as the tRNAs from GtRNAdb (30) and modified through the use of the CoCo correct annotation module (default parameters).

### Sequence similarity search

Each cluster sequence was aligned against each non-coding RNA sequence within the RNAcentral annotation (v10) (7) with nhmmer (31) (default parameters) to find sequence similarity. Hits with an *e*-value above 0.01 were filtered out. The overlap of the read clusters with the RNAcentral annotation was checked using the intersect tool from bedtools (parameters: -loj -s).

### Predictor parameters

Each cluster sequence was run through the following non-coding RNA predictors: snoscan (32) (parameters: -d 250 -l 1), snoGPS (33) (parameters: -t 105) and tRNAscan-SE (34) (default parameters).

TurboFold II (35) was used to find common structures between the new ncRNA genes and known tRNAs or H/ACA snoRNAs (using default parameters). Each Turbofold run combined the sequence of a new ncRNA gene with a set of 10 annotated tRNA or H/ACA snoRNA to compute common structure. The new ncRNA genes were marked as able to fold like a tRNA or an H/ACA snoRNA if they could adopt the reference structure of their respective sets. The two sets of 10 tRNAs and the two sets of 10 H/ACA snoRNAs used as references are listed in Supplementary Table S3. These sequence sets were selected for their ability to adopt the appropriate structure with Turbofold.

### Pol II and Pol III ChIP-Seq datasets and peak identification

Data for the ChIP-Seq of Pol II were obtained from (36): supplementary bigwig files GSE108323_ChIP_exoMerge.bw and GSE108323_ChIP_seqMerge.bw were downloaded from GEO series accession GSE108323. Data for the ChIP-Seq of Pol III originate from (37): supplementary bedgraph files from the compressed folder GSE18184_RAW.tar were downloaded from GEO series accession GSE18184. These datasets were visualized using IGV (38,39) and the locus of each non-annotated ncRNA was visually inspected for the presence of prominent ChIP-Seq peaks.

### Cell culture, DKC1 knockdown and tissue preparation

The ovarian adenocarcinoma SKOV3ip1 cell line was grown in DMEM/F12 (50/50) medium supplemented with 10% fetal bovine serum and 2 mM l-glutamine (Wisent) as recommended by ATCC. For the DKC1 knockdown, cells were seeded in six-well plates (350 000 cells/well) 4 hours prior to transfection with 10 nM DKC1 targeting siRNA using Lipofectamine 2000 (Invitrogen). Two replicates were performed using siRNA 1, targeting the third DKC1 exon (GAAUCCAAAGUUGCUAAGU) and two replicates were generated with siRNA 2, targeting the fourth DKC1 exon (ACACCUCUUGCAUGUGGUU). The cells were trypsinized and collected 72 h post transfection, pelleted and resuspended in 1 ml TRIzol (Ambion) and kept at −80°C until RNA extraction. Eight normal tissue samples: three prostate, two breast and three ovary (samples were obtained from FRSQ tissue bank (40)) tissue samples were homogenized with Tissuelyser and aliquoted in 1 ml TRIzol/30 mg tissue and also kept at −80°C prior to extraction.

### RNA extraction

Total RNA was extracted from transfected cells and their control (mock transfection), as well as from the tissue samples, with Qiagen's RNEasy minikit as previously described (16), with on-column DNAse 1 digestion. Samples were then evaluated on an Agilent Bioanalyzer.

### Library preparation for sequencing of RNA from human tissues and the SKOV3ip1 cell line

In order to prepare TGIRT-Seq libraries, 5 μg of each RNA sample, to which a 1 ul spike-in of ERCC EXfold mix 1 was added (ThermoFisher Scientific), were ribo-depleted using a RiboZero Gold (Human/Mouse/Rat) kit (Illumina). 50 ng of each RNA sample was then fragmented using the NEBNext Magnesium RNA Fragmentation Module (New England Biolabs) at 94°C for 2 or 3 min (depending on RIN) and treated with T4 polynucleotide kinase (Epicentre) to remove 3′ phosphates and 2′,3′-cyclic monophosphates, which impede TGIRT template switching (41). RNAs were purified after ribo-depletion, fragmentation and dephosphorylation using a modified version of the Zymo RNA Clean & Concentrator protocol (addition of eight sample volumes of ethanol to increase retention of very small RNA species). The recovered RNA was used for cDNA synthesis via TGIRT template switching with 1 μM TGIRT-III RT (InGex, LLC) for 15 min at 60°C, as previously described (16). The template-switching reaction seamlessly links the complement of an Illumina Read 2 sequencing primer-binding site (R2R DNA) to the 5′ end of the cDNA during cDNA synthesis, after which a DNA oligonucleotide containing the complement of an Illumina Read 1 sequencing primer-binding site (R1R DNA) is ligated to the cDNA 3′ end using Thermostable 5′ AppDNA/RNA Ligase (New England Biolabs). Ligated cDNAs with R1R and R2R sequencing adapters on either end were then amplified for 12 cycles of PCR (16) (initial denaturation at 98°C for 5 s, followed by cycles of 98°C for 5 s, 65°C for 10 s, 72°C for 10 s), during which Illumina capture and index sequences were added. Libraries were purified using Ampure XP beads (Beckman-Coulter) to remove adapter-dimers and leftover primers, then evaluated on an Agilent 2100 Bioanalyzer, and quantified using Qubit 2.0 fluorometer (Invitrogen) as was the final equimolar pool of the TGIRT-Seq libraries. They were then sequenced using the Illumina NextSeq 500 instrument (75 nt paired-end reads) with the Illumina NextSeq 500/550 High Output Kit v2 (150 Cycles). RNA-Seq data were processed and analysed as previously described (16,29).

### Conservation analysis

PhastCons100way score table for the hg38 assembly was obtained from the UCSC table browser (42,43). The conservation score per gene was calculated as the average phastCons100way score over all positions of the gene. The equivalent genomic coordinates of the non-annotated genes in 10 mammal genomes were obtained using the liftOver tool. Sequences were fetched from the extended equivalent coordinates (±300 nt upstream and downstream) of the mammalian genomes and aligned against the human non-annotated gene sequences with the matcher aligner tool from EMBOSS (44,45) to obtain identity percentage values.

## RESULTS

### Detection of non-annotated ncRNA genes

**Figure 1.**
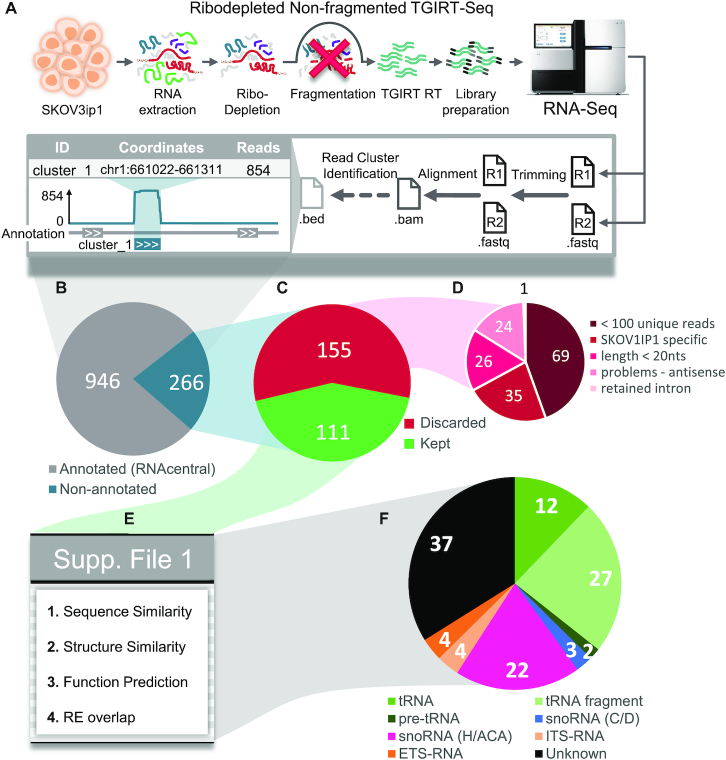
Identification of new non-coding RNA genes from sequencing read clusters. (**A**) Description of the RNA-Seq and bioinformatic analysis pipelines used for RNA detection and identification. The lack of fragmentation is indicated by an X. R1 and R2 indicate forward and reverse read files obtained from paired-end sequencing. Read clusters were identified using Blockbuster on bam alignment files, and visualized on a genome browser. (**B**) Identification of new non-coding RNA clusters. The RNA clusters obtained from A were compared to the annotation available in RNAcentral and clusters with and without standard annotations are indicated in the pie chart. (**C**) Proportion of kept and discarded clusters. Clusters with <100 uniquely mapped reads, a size smaller than 20 nucleotides, not detected in other investigated TGIRT-Seq datasets, poorly defined antisense fragment of the 45S rRNA or representing a retained intron were discarded. It should be noted that clusters with fewer than 100 uniquely mapped reads but with multimapped reads only aligning to other non-annotated clusters were kept (such is the case for the ETS and ITS clusters). (**D**) Distribution of the discarded clusters non-optimal features. (**E**) Methods used for the functional (biotype) classification of the new non-coding RNA genes. The retained clusters were compared to the sequence, structure, function and repeated element overlap of known ncRNA and classified by biotype. (**F**) Final distribution of the predicted biotype of the NA_RNA clusters.

To identify non-annotated ncRNA genes, ribodepleted non-fragmented RNA extracted from the ovarian cancer cell line SKOV3IP1 was sequenced using TGIRT-seq (Figure 1A), which enables full length sequencing of RNA varying between 20 and 500 nucleotides (nts) in length (16). We avoided RNA fragmentation to obtain a better definition of the exact start and end of the detected ncRNA and, as such, facilitate *de novo* transcript annotation. As shown in Supplementary Figure S1, sequencing reads generated using this method have more uniform read alignment profiles than the standard fragmented RNA-Seq (16). To identify potential ncRNAs from sequencing read profiles, we used the Blockbuster software (26), which detects clusters of overlapping reads (Figure 1A). Read clusters smaller than 500 nts and composed of at least 100 reads were selected, and those not overlapping previously known annotations, as defined in the all-inclusive database RNAcentral (v10) (7), were identified using Bedtools intersect (27) (Figure 1B). RNA in RNAcentral obtained from sources that do not provide genomic coordinates such as ENA (46), or that are only present in a single specialized database with little experimental validation such as snoRNA Atlas (47) and piRNABank (48) entries were considered previously non-annotated. To increase confidence in the newly detected RNA, low abundance clusters found in the antisense of 45S ribosomal RNA, fully retained introns, those with less than 100 uniquely mapped reads and / or detected in a single sequencing dataset were filtered out (Figure 1C, D). Figure 1D shows the number of clusters removed using these filters. As a result of this pipeline, out of the 1212 read clusters detected by Blockbuster, we identified 111 (9%) robustly expressed previously non-annotated transcripts that we termed non-annotated RNA or NA_RNA (Figure 1C). We note that the majority (64/111) of NA_RNA have only uniquely mapping reads and 93% (103/111) of NA_RNA have <20% of reads also mapping to other already annotated genes. NA_RNA were not restricted to a specific chromosome or locus but were distributed throughout the genome. Together the data demonstrate the advantage of using an annotation-independent analysis pipeline in these specialized RNA-seq datasets and indicate that the annotation of the human genome is not complete even when the most general of gene annotations are used.

### Profiling and characterization of the NA_RNAs

Four main analyses were used to determine the likely biotype and possible function of the NA_RNAs: (i) sequence similarity analysis, (ii) family-specific structure prediction, (iii) function prediction and (iv) the identification of overlap with repeated elements (Figure 1E and Supplementary File 1). Sequence homology searches using nhmmer (31) identified 69 NA_RNA with homology (*e*-value < 0.01) to known ncRNA biotypes. Of these 69 NA_RNA, 41 displayed homology to tRNA, 20 to snoRNA, snRNA, 7SL RNA or rRNA and eight exhibited homology to antisense RNA or lncRNA. Surprisingly, 32 NA_RNAs had no significant similarity with any known transcript in RNAcentral indicative of potentially new classes of ncRNA. In parallel to the sequence homology searches, family-specific structure prediction was performed using Turbofold (35), which makes an iterative probabilistic estimate of secondary structures. By using 40 reference tRNAs and H/ACA snoRNAs, we identified 11 NA_RNAs that fold like tRNA and 21 that fold like H/ACA snoRNAs. Function prediction of the NA_RNA sequences was also carried out, using ncRNA predictors snoGPS (33) for H/ACA snoRNAs, snoscan (32) for C/D snoRNAs and tRNAscan-SE (49) for tRNAs. The snoGPS and snoscan predictors identified seven NA_RNAs as H/ACA snoRNA and three as C/D snoRNAs respectively. The tRNAscan-SE predictor did not identify any of the putative tRNAs identified through sequence and structural homology, most likely due to small structural deviations from the canonical tRNA model, as discussed later. Comparing NA_RNA to known repeat elements using RepeatMasker (50,51) identified 18 NA_RNA in tRNA repeats and 26 within Alu repeats, half of which have been identified as ‘Alu H/ACA snoRNA’ by the database snoRNA atlas including eight Alu snoRNA with similarity to 7SL RNA (47) (Supplementary Table S4). Six of the predicted H/ACA snoRNAs shared similarity to antisense RNA or lncRNA and eight to 7SL RNA.

Following these independent and complementary analyses, we then integrated their results to obtain the classification of the 111 NA_RNAs into 8 functional classes including tRNA-like, pre-tRNA, tRNA fragment, C/D snoRNAs and H/ACA snoRNAs, ITS-RNA, ETS-RNA and unknown RNA (Figure 1F, Supplementary File 1). The tRNA and snoRNA classes comprise NA_RNAs with the capacity to fold as tRNA or snoRNA and/or have sequence homology to these biotypes and/or have been predicted by function predictors. The tRNA fragment class includes NA_RNAs with sequence homology to tRNA genes but which are too short (<65 nt) to form a canonical tRNA structure, while pre-tRNA are defined as RNA fragments with sequence homology extending beyond the established mature tRNA sequence. The ETS-RNA and ITS-RNA include RNA aligning to the 5′ External Transcribed Spacer (ETS) and the Internal Transcribed Spacer (ITS) of the 45S preribosomal RNA, respectively. The rest of the NA_RNAs are grouped as unknown. We conclude that the majority of non-annotated read clusters presented here are *bona fide* ncRNAs including tRNA and snoRNA that were previously missed due to detection biases from classical RNA-Seq methodology and/or poor homology.

### Non-fragmented TGIRT-seq enhances the detection of the NA_RNAs

**Figure 2.**
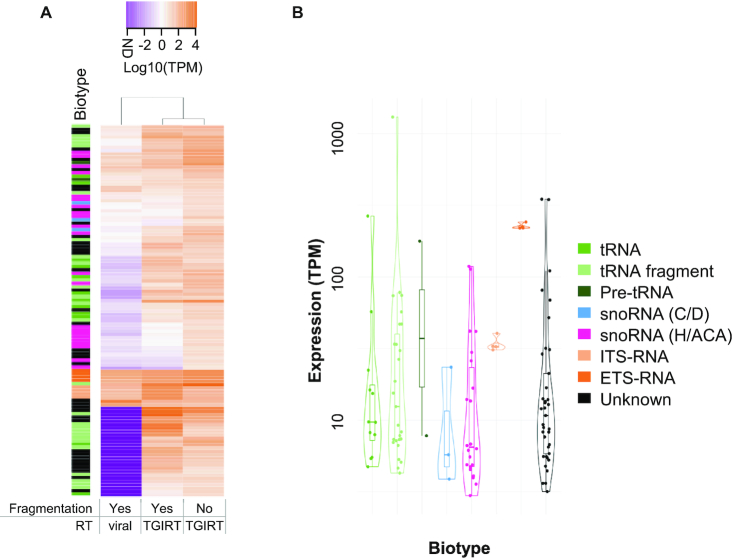
Detection of NA_RNA depends on the sequencing method. (**A**) Viral based sequencing library protocols poorly detect most of the NA_RNA. The abundance of 111 NA_RNA in three different RNA-Seq library preparation methods was compared and the unsupervised clusters shown in the form of a heatmap. All sequencing methods were generated using ribo-depleted SKOV3ip1 RNA. Color legend for abundance is given at the top (ND: not detected). The sequencing methods are indicated at the bottom. Gene biotype colors are indicated on the right of panel B. (**B**) Violin plots of the distribution of NA_RNA abundance as detected in non-fragmented SKOV3ip1 datasets are shown for each biotype. The color legend for biotypes is on the right.

It was previously shown that non-fragmented TGIRT-Seq offers an excellent sampling of ncRNAs with low structure bias (16). This capacity to detect structured RNA increases the likelihood of detecting NA_RNAs as indicated in Figure 2A. Many NA_RNAs, and especially NA_tRNA (non-annotated tRNA) and NA_snoRNAs (non-annotated snoRNAs), were missing or detected at very low level in viral reverse transcriptase sequencing datasets and clearly detected by TGIRT-seq (Figure 2A). RNA treatment also affects the detection of NA_RNA. Skipping RNA fragmentation improves the definition of transcript termini, provides more uniform alignment profiles and enhances the detection of NA_RNA by reducing the number of reads generated from long RNAs like mRNAs and lncRNAs (Supplementary Figures S1 and S2A). Therefore, non-fragmented TGIRT-Seq is the best approach to detect NA_ncRNA. Using this method, we detect NA_RNA with variable abundance depending on the biotype, the most abundant being the tRNA fragment group and the least abundant being the snoRNA (C/D) group (Figure 2B). The top four most expressed NA_RNAs were the NA_tRNA_fragment cluster_1088, unknown RNA cluster_177, unknown RNA cluster_1065 and NA_tRNA cluster_5 scoring 500–1000 TPM (transcript per million). However, in general the majority of NA_RNA were expressed below 100 TPM. Notably, as indicated in Supplementary Figure S2, the abundance of NA_RNA was well within the expected expression level for their biotype, which further confirms the NA_RNAs biotype identity. Together these data suggest that the majority of NA_RNA are difficult to detect using standard sequencing techniques given their stable structure and small size (Supplementary Figure S3), which may explain their absence from genome annotation.

### NA_RNA expression is not restricted to a single cell type

Gene expression is largely deregulated in cancer cells and often replete with cryptic transcripts which may explain the identification of *de novo* transcripts (52–54). To evaluate this possibility, we examined the expression of the NA_RNAs in different tissues using both in house and published fragmented TGIRT-seq datasets. A total of 15 samples from different normal and cancer tissues as well as normal immortalized and cancer cell lines were considered including normal breast, brain, ovary and prostate tissues and the expression of the NA_RNAs compared. Since many of the NA_RNAs were found in introns or within repeat clusters, sequencing was performed using fragmented RNA in order to evaluate possible links to the expression of host genes. All 111 NA_RNAs were detected in datasets coming from tissues or cell lines other than SKOV3ip1, indicating that the newly identified genes are not anomalous products of diseased cells, but rather *bona fide* genes missing from the current genome annotations (Supplementary Figure S4). Notably, while some of the NA_RNAs, like the tRNA, are highly and generally uniformly expressed across most datasets, others and in particular those that do not resemble known ncRNA (unknown) and H/ACA snoRNA are of lower and more variable expression across most tissues. As observed in the ovarian cancer cell line SKOV3ip, most NA_RNAs were found to be moderately expressed with an abundance varying between 5 and 10 TPMs, although a few tRNAs are expressed with >100 TPMs (Figure 2B). Comparing the expression level across different tissues indicates that the strongest differences in abundance of the NA_RNAs are found in brain tissue samples and in the human reference RNA sample (Supplementary Figure S4). The most homogeneously expressed group of NA_RNAs across the different tissues are the tRNAs, which is expected for this highly conserved class of RNA. We conclude that missing annotations are generally not due to tissue specificity or generalized low expression level and that NA_RNAs represent a robust group of mostly homogeneously expressed genes.

### NA_RNAs are expressed in a biotype-dependent manner

To investigate how the NA_RNA genes are expressed and further validate their biotype attribution, we started by characterizing the genomic context in which they are encoded. Most annotated human snoRNAs are intronic, encoded in the introns of either protein-coding or long-non-coding RNA host genes (55,56). As shown in Supplementary Figure S5A, all 25 NA_snoRNA are also intronic, embedded in longer annotated genes. In contrast, a majority of NA_tRNA, NA_tRNA_fragment and NA_pre-tRNA are intergenic as is the case for annotated tRNAs (10,11). As expected, ITS and ETS derived NA_RNAs are embedded in the rRNA clusters, while the unknown NA_RNAs have a mixed genomic context, consistent with their presumably mixed biotype. We also consulted previously published Pol II and Pol III ChIP-Seq datasets (36,37) in an attempt to determine polymerase dependency. Using these datasets, overlapping polymerase peaks were found on only nine NA_RNA clusters for Pol III and only one for Pol II (Supplementary Figure S5B), suggesting many NA_RNA are not independently transcribed. However, the antisense or intergenic context of many NA_RNAs implies that they must be independently transcribed in some manner. Therefore, at least for these seemingly independent genes, the low number of peaks might stem from variation in the cell lines used in the different studies and/or the lack of sufficient sequencing depth to detect their low abundance level. On the other hand, absence of distinct Pol II peaks from intronic NA_RNAs is expected since they are transcribed as part of the host gene expression. Consistently, expression of these intron embedded NA_RNAs correlates positively with the expression of their host genes (Supplementary Figure S6). Such host-dependent transcription is known to occur for most known human snoRNAs (56) and our results suggest that this expression mode is used by at least 21 NA_snoRNAs as well as 8 intronic unknown NA_RNAs (Supplementary Figure S5B and S6A, B). ETS and ITS RNAs are a special case in that they are nested in the ETS and ITS1 of the Pol I transcribed 45S rRNA. Correlation between these RNA and their host rRNA gene expression cannot be verified since most rRNA are absent from the ribodepleted RNA-Seq dataset used in this study. Having no Pol II or Pol III peak to support independent expression of the rDNA embedded NA_RNA, we speculate that they are produced from the processing of the 45S rRNA spacers. Ultimately, most NA_RNAs appear to be independently transcribed, whether because they have an associated polymerase ChIP peak, because they are intergenic or antisense, or because their expression level is negatively correlated with that of their host gene (Supplementary Figure S5B and S6C, D). Most importantly, NA_RNA followed the expression pattern of their respective attributed biotype, most NA_tRNA being independently transcribed while most NA_snoRNA generated from introns being correlated with the expression of their host genes, once again confirming their identity.

### NA_RNAs exhibit biotype specific conservation and genomic distribution patterns

**Figure 3.**
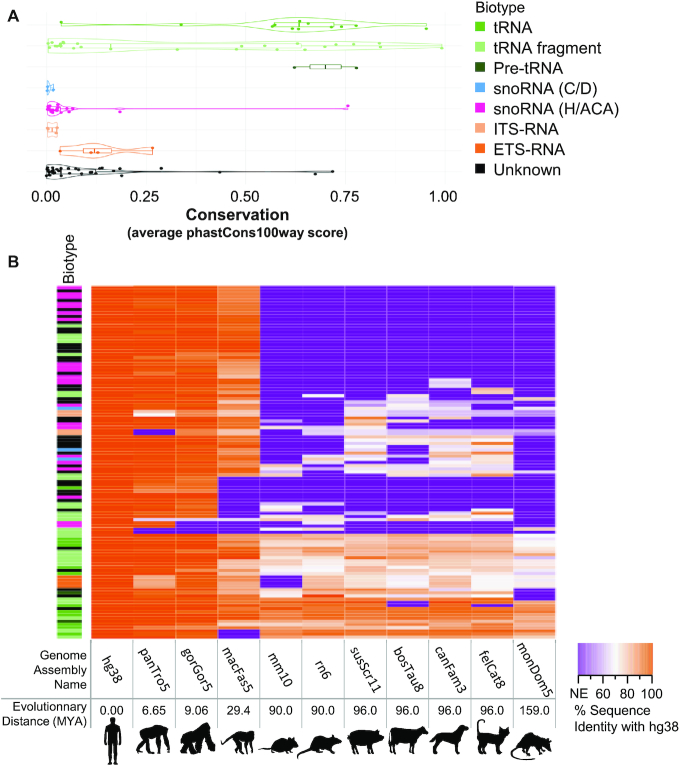
NA_RNA display biotype dependent conservation patterns. (**A**) Violin plots of the distribution of the conservation level of each NA_RNA as determined using the phastCons100way score (average of the score for each nucleotide position) by biotype (color legend on the right). (**B**) The conservation of each NA_RNA in mammals is presented as a heatmap based on the identity score of the closest hit. The color index (% sequence identity with human) is shown on the bottom right. The absence of an equivalent gene (NE: no equivalent) is shown in purple while orange indicates a highly similar or identical sequence. The 11 mammalian species used for the comparison are sorted in ascending order of evolutionary distance from human and the distance estimated by timetree.org in millions of years (MYA) is shown at the bottom. The biotype of the NA_RNAs is indicated on the left.

To investigate the evolutionary origin of the NA_RNA genes, we calculated their overall conservation levels as given by the average phastCons100way score, which measures the odds that nucleotides at each position of a sequence are conserved in 100 vertebrates (42). To complement this analysis, we also searched for orthologs of these genes in 10 mammalian species and investigated their sequence identity levels. As expected, the most conserved group of NA_RNAs are the tRNAs, tRNA fragments and pre-tRNAs, with most tRNA conserved at least as far as the opossum (*Monodelphis domestica*) (Figure 3). This is to be expected as tRNAs are the most highly conserved ncRNA class across all domains of life (57). The conservation level of the NA_tRNAs and NA_pre-tRNAs is typical of that of annotated tRNAs, while the conservation of NA_tRNA fragments tends to be lower (Supplementary Figure S7A). Interestingly, NA_tRNA fragment cluster_1153 has no orthologs in other species and could, therefore, be a human-specific gene.

The NA_snoRNAs are generally poorly conserved outside primates and have a low PhastCons100way score compared to known snoRNAs (Supplementary Figure S7B). Unsurprisingly, all NA_H/ACA snoRNA overlapping Alu elements are only found in primates, as Alu elements are unique to primates (58). The fact that most NA_snoRNAs are associated with repeated elements and have very low conservation levels likely indicates that they correspond to evolutionarily recent transposition events and fits well with previous models of snoRNAs spreading throughout genomes recently thanks to their relationship with transposable elements (59). In fact, amongst the novel genes, only one C/D and two H/ACA NA_snoRNA do not overlap a retrotransposable element. It is important to note that none of these snoRNA-like clusters show high sequence similarity to known snoRNAs. This might mean that these genes have originated from snoRNA copies that have rapidly evolved due to functional redundancy or that they were fortuitously produced by the mutation of intronic elements, including retrotransposons.

The ITS and ETS-RNA groups both have a poor sequence conservation level, which corroborates the known high sequence variability of these pre-rRNA spacer regions (60). While most of the NA_RNA genes from the ‘Unknown’ group have low conservation levels, a few such as cluster_675, are very highly conserved. No correlation is found between the expression level and the conservation score for any of the NA_RNA groups, with some of the most expressed being poorly conserved and vice versa. The high conservation and low expression of some of these NA_RNAs could be explained by tissue- or stress-specific expression patterns. On the other hand, some NA_RNA genes are highly expressed but have a low conservation score. These include the most expressed tRNA fragment (cluster_1088), the most expressed unknowns (cluster_1065 and cluster_177) and the four ETS-RNAs. It is possible that these genes are important contributors to primate cells or represent human specific features. Overall, the conservation and genomic distribution patterns of NA_RNAs strongly support their biotype classification.

### The NA_tRNA adopt the transcription and folding patterns of tRNAs

**Figure 4.**
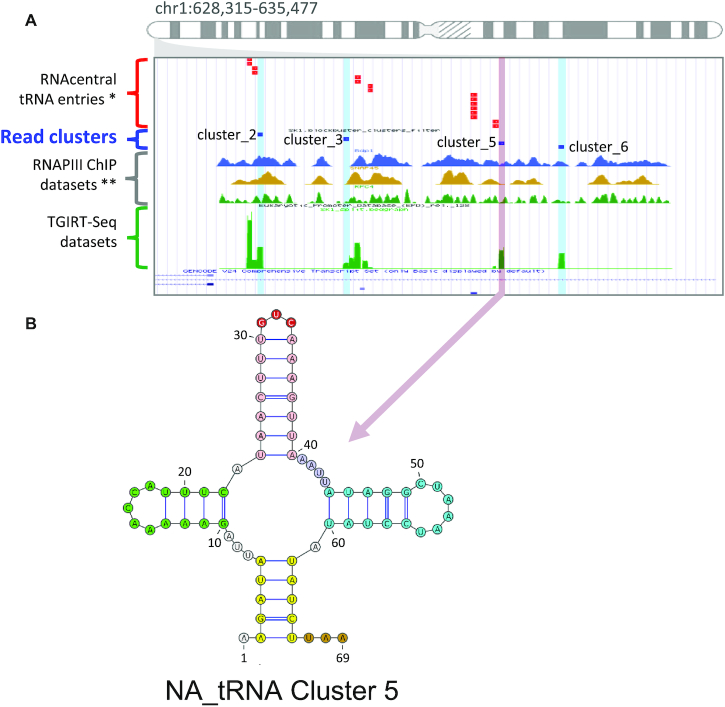
The NA_tRNAs exhibit the structural features of known tRNAs. (**A**) Genome browser view showing the location of a newly identified tRNA (cluster 5, indicated in magenta) near three additional non-annotated multimapped loci (highlighted in blue – not part of the 111 NA_RNA genes) and previously annotated tRNAs (in red). (**B**) Predicted secondary structure of cluster_5. The secondary structure was predicted using turbofold and the structural features of tRNAs highlighted in colors (the acceptor stem, D arm, anticodon arm available loop and T arm are shown in yellow, green, magenta and blue, respectively while the anticodon is indicated on top in red).

To further confirm the identity of the NA_tRNA, we compared their expression mechanism and folding pattern to that of known tRNAs. Five of the 8 NA_RNAs overlapping a Pol III ChIP-Seq peak as discussed above are classified as tRNAs, tRNA fragment or pre-tRNA. The strongest enrichments of RNAPIII ChIP-Seq peaks were observed within a cluster of tRNAs at the beginning of chromosome 1 that holds 4 NA_tRNAs residing near six previously annotated tRNAs (Figure 4A). Three of these four clusters happen to be identical in sequence to other tRNA genes and consequently were discarded by the multimapping filter (Figure 1C, D) and they are not included within the selected 111 NA_RNAs. However, their obvious transcription by pol III and their close proximity to other tRNAs are strong indicators that they too are NA_tRNA genes. We show that cluster_5 can also adopt a tRNA-like structure although it holds some non-canonical features (Figure 4B, Supplementary Figure S8).

Indeed, even though Turbofold has shown that 6 of the 12 NA_tRNAs can properly fold in a three-leaf clover structure, they all have imperfections when compared with the canonical tRNA model, explaining why none were predicted as being tRNAs by tRNAscan-SE. For example, cluster_5 has an anticodon stem of 7 bp, longer than the expected 5 and has a very small anticodon loop (Supplementary Figure S8). It is possible that these tRNA-like genes have evolved specialized functions. In any case, six of these NA_RNA genes share close structural resemblance with tRNAs and most probably are evolutionarily linked to tRNAs. For the other six tRNA-like NA_RNAs that do not seem to be able to adopt a proper tRNA structure even though they are highly similar in sequence, their function is more elusive. For that reason, we would classify them as pseudo-tRNA genes.

To further validate these tRNAs, we analysed datasets of alternative RNA-Seq methodologies that are specialized in the detection of tRNAs to see if they detect our NA_tRNAs. We analysed datasets from the hydro-tRNASeq methodology (18), the YAMAT-Seq methodology (19) and the Demethylase TGIRT-Seq methodology (20). Of the 12 NA_tRNAs, all are detected by at least one of these alternative specialized tRNA methodologies, and 9/12 are detected by all three approaches (Supplementary Figure S9). Overall, the sequence homology, expression pattern and genomic organization support the proposed biotype of these NA_tRNA despite their modest deviations from the features of the canonical tRNA structure. It is interesting to note that all but one of the 12 NA_tRNA overlap so-called NumtS (Nuclear mitochondrial sequences) which are mitochondrial fragments inserted in the nuclear genome (61). The NA_tRNA are much shorter than the overlapping NumtS regions. Thus we conclude that our approach detects short regions of NumtSs as strongly expressed. The special features of these NA_tRNAs might be indicative of alternative or specialized functions.

### The tRNA fragments originate from non-annotated tRNAs and tRNA-like short RNAs

**Figure 5.**
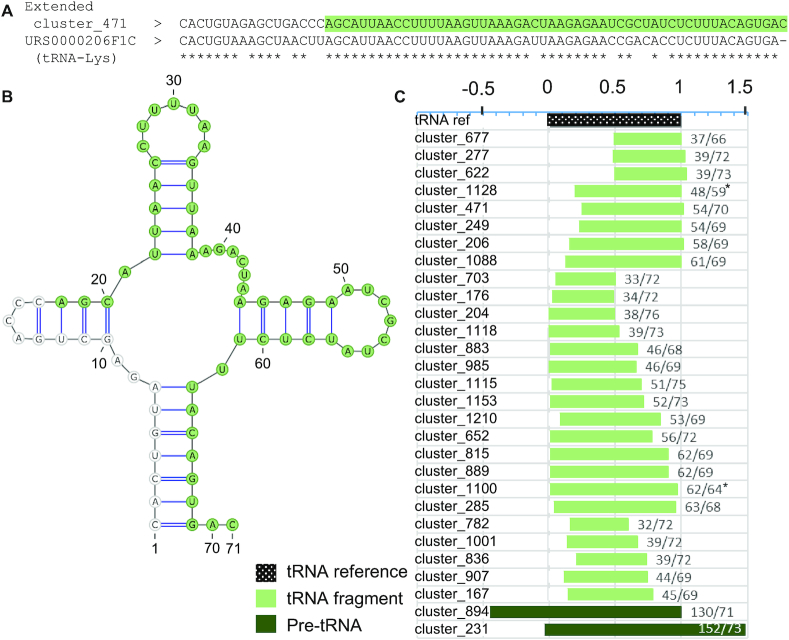
The newly identified tRNA fragments are generated from canonical tRNA genes. (**A**) Alignment of the sequence of cluster_471 with the sequence of its most similar tRNA gene (tRNA-lys URS0000206F1C). Identical nucleotides are indicated by asterisks and mismatches by blank spaces; the fragment sequence is highlighted in green. (**B**) Predicted structure of the RNA fragment cluster_471 in the context of the mature RNA of its reference gene. The fragment sequence is highlighted in green. (**C**) Distribution of the size and position of the NA_tRNA fragments relative to their reference genes. 0 and 1 indicate the mature 5′ and 3′ ends of the reference gene, respectively. The ratio of fragments over reference gene length is indicated on the right. The asterisk indicates incomplete reference tRNA that may not fold into mature tRNA structure.

Although they were not aligned to specific tRNA genes and were too small to be considered as tRNAs, 27 of the NA_RNAs share high sequence similarity with tRNAs elsewhere in the genome and many of them are overlapping tRNA gene repeats identified by RepeatMasker, and so we classified them as tRNA fragments (tRFs) (Figure 1F, Supplementary File S1). To assess whether these tRFs are indeed produced from the processing of a non-annotated tRNA gene, we extended the fragment sequence to be equivalent in length and position to their reference tRNA (the most similar tRNA sequence found in RNAcentral). While cluster_167, cluster_249, cluster_471, cluster_815 and cluster_1115 have >75% sequence identity with their reference tRNA in their extensions, all the other tRFs have low sequence similarity with their reference tRNA in their extension (Supplementary File 2). The same Turbofold assay used to identify the tRNA clusters shows that the extended forms of cluster_167, cluster_204, cluster_471, cluster_815 and cluster_985 can fold in a similar fashion to tRNAs although the size of their different stems is, once again, inappropriate in some cases (Figure 5, Supplementary Figure S10). Finally, read alignment profiles from the non-fragmented SKOV3ip1 datasets as well as the structure of extended fragments show that some of these tRFs appear to be expressed together with a full size tRNA transcript, giving good confidence that these transcripts are produced from the nucleolytic processing of a non-annotated tRNA (Supplementary Figure S10, S11). Investigation of the profiles in the different tissue datasets did not show any significant difference in the read alignment profile shape. Although this evidence indicates that some of these NA_RNA genes are fragments produced from non-annotated tRNA genes, most of these NA_RNA genes are only assigned to this class due to their sequence similarity to tRNA. They may be genes with no functional tie to tRNA as in some cases, their extensions seem inappropriate to produce a functional tRNA.

tRFs are known to come from different portions of their tRNA gene and so they have been divided in different groups in the literature such as 5′ and 3′ halves, 5′ and 3′ fragments, and internal fragments (62,63). To determine to which tRF class these fragments belong, we looked at their alignment position with respect to their reference tRNA (Figure 5C). Although 4 clusters appear to be 5′ halves, 3 are 3′ halves and 5 are internal fragments, the rest are much harder to fit in previously established categories. Indeed, the rest appear to be ¾ or close to full tRNA fragments that are slightly too short to fold like tRNAs. Also, the NA_tRNA fragment cluster_1118 overlaps a piRNA sequence entry in piRNABank. This corroborates previous findings that tRNAs can produce piRNAs through processing (64). Overall, the data underscore the broad spectrum of tRNA-derived transcripts and suggest that the human transcriptome is replete with expressed tRNA-derived short transcripts awaiting functional characterization.

### NA_snoRNAs associate with snoRNP proteins and target known rRNA modification sites

**Figure 6.**
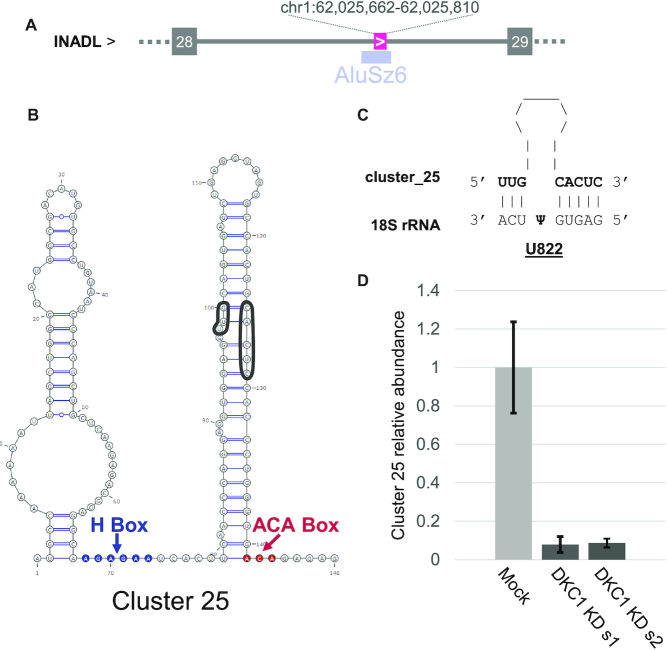
The NA_H/ACA snoRNA are part Alu repeat elements. (**A**) The position of NA_H/ACA snoRNA cluster_25 (magenta box) is shown relative to the exons of its host gene (gray boxes) and the intronic AluSz6 element (blue box). (**B**) cluster_25 adopts the structure of an H/ACA snoRNA. The structure was predicted by both Turbofold (35) and snoGPS (33) showing the position of the H and ACA boxes. The snoRNA target-binding sites are circled. (**C**) The predicted modification target of cluster_25 in the 18S rRNA was determined using snoGPS and the pairing between the snoRNA and target indicated by the black lines. (**D**) Depletion of the H/ACA snoRNA binding protein dyskerin inhibits the expression of cluster_25. The average RNA abundance in TPM was determined in sequencing datasets of lipofectamine controls and cells transfected with two independent siRNAs targeting DKC1 (s1 and s2).

The three C/D and seven H/ACA snoRNAs identified by snoGPS (33) and snoscan (32) exhibit the canonical features of snoRNA, appropriate box C/D and H/ACA positioning and identified ribosomal RNA modification targets (Supplementary Table S4). NA_snoRNA ribosomal targets are evenly spread throughout the 18S and the 28S (Supplementary Table S4). All of these targets are shared with other snoRNAs, but the NA_snoRNAs display no significant sequence identity with the annotated snoRNAs mediating the same modifications. This is not surprising as the target position of snoRNAs can be interchangeable through mutations of the guide sequence, and so, similar snoRNAs can have different targets just as dissimilar snoRNAs may share the same targets (56,65). Structure prediction with Turbofold and box considerations identified an additional 12 H/ACA snoRNA-like NA_RNAs that were not predicted by snoGPS for a total of 22 H/ACA NA_snoRNAs. These additional 12 H/ACA NA_snoRNA genes have no rRNA target predicted by snoGPS and so they can be classified as orphans. Very few orphan H/ACA snoRNAs are annotated and little interest have been shown for their potential for non-canonical functions which could be explained by the difficulty in detecting these RNA using standard sequencing techniques (16). Fourteen of these orphan NA_H/ACA snoRNA and four of the NA_snoRNAs with rRNA targets are Alu snoRNAs, which is a class of snoRNA embedded in Alu repeats. Ten of the orphan Alu snoRNAs were identified in a previous study as Wdr-79 associated box H/ACA RNPs (66), and are present in the snoRNA atlas (47) but were not incorporated in the Ensembl or RefSeq databases. These Alu snoRNAs feature atypical 5′ stem loop size and their involvement in rRNA pseudouridylation remains unverified (67). However, target prediction suggests that four of these NA_Alu snoRNAs may target rRNA modification (Figure 6, Supplementary Table S4). To confirm the identity of NA_H/ACA snoRNA, we examined the impact of depleting the H/ACA binding protein dyskerin on the abundance of NA_H/ACA snoRNA. As indicated in Figure 6D and Supplementary Figure S12, the knockdown of dyskerin reduced the abundance of NA_H/ACA snoRNA to the same level as annotated H/ACA snoRNAs without affecting C/D box snoRNA. Together these data confirm the accuracy of our NA_RNA biotype assignment and validate the prediction of a new group of previously non-annotated H/ACA snoRNA genes.

### The pre-rRNA spacer sequence generates discrete structured stable transcripts

The four ETS-RNA NA_RNAs and four ITS-RNA NA_RNAs are held within four respective copies of the 45S rDNA (Supplementary Figure S13A). The sequences of the ETS-RNA transcripts are identical, although cluster_731 is shorter than the others by four nucleotides. The same is true for the ITS-RNA transcripts, being identical in sequence, with short differences as to where the transcripts begin and end. For that reason, very few of the reads overlapping these eight regions are uniquely mapped (between 0.5 and 10% of reads per locus are uniquely mapped, the other reads being shared with the other three RNAs, but not with any other genomic locus) and so it cannot be determined if these four ETS and four ITS derived transcripts are all strongly expressed, but some of them certainly are. Nevertheless, these transcripts accumulate in considerable abundance, especially for the ETS-RNA which all have an estimated abundance of ∼400 TPM. Interestingly, the ETS-RNA NA_RNAs presented here are very close in position to the 5′ end of the 5′ ETS and are next to two annotated genes of very similar size, present in all four 45S loci: MIR6724-(1 to 4) and a NoRC associated RNA. No sequence similarity was found between these three genes but Turbofold and RNAfold show interesting structural similarity between the three as they can all form a long single stem structure (Supplementary Figure S13B). The ITS NA_RNAs, on the other hand, appear to be alone in the ITS1 region, with no annotated stabilized transcripts. Their most striking feature is the highly repetitive nature of their sequence, especially at the 3′ end, which is particularly GA rich (Supplementary Figure S13A). These four ITS1 derived copies are situated between the cleavage sites 2 and 3 and are, therefore, likely a stabilized fragment of the normally degraded segment between the two sites. Indeed, we did not detect independent promoters or transcription initiation sites near the termini of these NA_RNA further supporting their generation from pre-rRNA processing intermediates.

### NA_unknown RNAs include RNA fragments, retrotransposons and potential new RNA families

The Unknown NA_RNA family comprises genes of a wide range of sizes, expression levels and conservation levels and lacks significant sequence similarity with other genes. Sequence similarity searches did not identify any significant similarities with *e*-value < 0.01 mostly due to the short cluster size (<30 nts). In nine cases, the RNA shared sequence similarity with short portions of tRNAs, although they are too short to be full-size functional tRNAs. Other unknown NA_RNAs share sequence similarity with a small portion of an annotated gene but they are too short to form a functional copy of this gene. For example, cluster_273 displayed similarity to the 3′ third of the U12 minor spliceosomal RNA gene and cluster_739 displayed similarity to the 5′ half of the SNORA33 H/ACA snoRNA gene. Interestingly, extension of these fragment sequences shows poor sequence similarity with the reference RNA. Fourteen other RNA in the group did not share sequence similarity with any known gene but were in the size range of miRNAs or piRNAs (Supplementary Figure S3). However, the biggest group (27/37) of the unknown clusters overlap with a portion of retrotransposon elements (Supplementary Figure S14), which might be indicative of retrotransposons that are still being partly transcribed. For the rest of these ‘Unknown’ clusters, not much can be said about their potential functional family due to their short size and lack of significant homology. Together, this work indicates that the repertoire of stable small RNA is far from complete and that the human transcriptome is replete with small RNA fragments that await annotation, as well as mechanistic and functional characterization.

## DISCUSSION

We have previously shown that ribodepleted non-fragmented TGIRT-Seq allows the detection of a much wider range of non-coding RNAs than standard RNA-Seq (16). Here, we show that this technique detects RNAs that have not yet been annotated. Their lack of annotation is either due to the fact that they are generally hard to detect with classic sequencing methods and/or that they imperfectly fit in characterized RNA families due to non-canonical features. Indeed, it certainly is not a coincidence that most of these 111 non-annotated regions are predicted as either tRNA-like or H/ACA snoRNAs, two highly-structured RNAs we have previously shown to be greatly under-represented in classic RNA-Seq datasets (16). Also, many of these non-annotated genes have imperfections in relation to their predicted RNA families. However, in most cases tested, the expression and conservation patterns, the structure, the genomic location, and the response to protein depletion validated the newly predicted gene biotypes as bonafide new genes with a robust expression level. Indeed, most of the NA_RNA genes were expressed in a wide variety of tissues and a few were expressed at very high level (Figure 2, Supplementary Figure S4). Their genomic location and expression levels mirror those of their assigned biotype (Figure 2, Supplementary Figure S3–S6), as do their structure and conservation patterns, confirming their assigned biotypes (Figures 3, 4 and Supplementary Figure S7–S8). Finally, biotype-specific experimental evidence such as tRNA-seq and dyskerin depletion further support the classification (Figure 6, Supplementary Figures S9 and S12). Together the work presented here underscores the importance of annotation-independent analysis of sequencing data and adds dozens of new experimentally validated genes to standard annotations.

The newly identified genes may represent a new class of RNA with alternative function. For example, we have found that some of the NA_tRNAs have stems or loops of inappropriate sizes, mismatches in stems or are simply unable to adopt the 3-leaf clover structure. Because of this, they are not positive hits for the tRNA predictor tRNAscan-SE. This explains why they have not been annotated as tRNAs by GtRNAdb (30) which uses tRNAscan-SE positive hits to identify tRNA genes. Although there are no tRNAscan-SE positive hits in the novel genes we describe here, most of them are highly conserved and expressed, and show strong sequence similarity to tRNAs and are detected in tRNA sequencing methodologies. Further validations would be necessary to determine whether the standard definition of the tRNA family should be extended to include these atypical tRNA genes. It is, however, entirely possible that some, if not all, of these NA_tRNA genes have distinct functions compared to canonical tRNAs, whether it be to produce fragments in specific conditions or to function in completely different cell pathways. On the other hand, the non-annotated tRFs come from all parts of tRNAs and range in size from 32 to almost full tRNA of 62 nucleotides (Figure 5). This challenges the notion that tRFs are necessarily limited to strict categories including 5′ fragments, 5′ halves, 3′ fragments, 3′ halves and internal fragments (62,63) and might be indicative of a much more complex family of transcripts. It is important to note that, not all of these tRFs may be produced from non-annotated tRNA genes. Indeed, our alignment results show that, although some might be expressed as full functional tRNAs in other cell types, others show no sequence identity with tRNAs in their extensions and cannot fold as tRNAs. Two likely models might explain these isolated tRFs. First, it might be possible that the redundancy of tRNA genes led to the evolution of loci that are only used to produce tRFs and so their upstream and downstream sequences quickly degenerated, losing the ability to produce a functional tRNA. Another hypothesis is that some of these tRFs were produced from a different tRNA gene locus and were captured by retrotransposable element machinery and then inserted somewhere else in the genome, as has occurred for snoRNAs (59). Overall, these results seem to indicate that tRFs are not just products of the processing of tRNA but might have become independent genes expressed on their own.

The newly identified snoRNAs described here fuel the debate about the alternative function of snoRNAs outside the documented role in rRNA modifications. While many of these RNA have clear modification targets, several others have no predictable rRNA target or are found in Alu repeat elements, which define a group of H/ACA snoRNA with unclear function. However, regardless of target predictability, all new H/ACA snoRNA behave like *bona fide* snoRNA. Their genomic distribution, folding and expression patterns fit that observed for known snoRNAs. Most importantly, these new snoRNA are sensitive to the depletion of the H/ACA RNA binding protein dyskerin confirming their H/ACA vocation. Notably, our work suggests that Alu H/ACA which were recently described as a distinct class of snoRNAs (67,68) may behave like canonical snoRNA. They are equally sensitive to dyskerin depletion supporting their nucleolar localization and pseudouridylation functionality. Confirming the link to Alu H/ACA snoRNAs, we identified CAB boxes in our newly identified Alu H/ACA, supporting previous evidence of an Alu H/ACA Cajal body-relationship (67). Interestingly, the Alu H/ACA snoRNAs are not always aligned on the same position relative to Alu elements, being produced both from Alu monomers and dimers. Together with the evidence of canonical function, this suggests that Alu H/ACA are not a functionally distinct class, but rather a fortuitous production of Alu repeats. This opens the possibility that any inclusion event might produce a stable transcript, provided a few mutations occur to make the H and ACA boxes. This relative simplicity might explain the large number of H/ACA snoRNA genes found in the genome and suggests that many other H/ACA snoRNAs might be awaiting discovery in other cell lines.

The structural similarity and genomic proximity of ETS-RNA to the MIR6724, a miRNA with no known target, and a NoRC associated RNA might offer clues as to their purpose. Indeed, these NoRC associated RNA are known to be recognized by TAM domain of BAZ2A (also known as TIP5), a component of the NoRC chromatin remodeling complex (69). These NoRC associated RNA have been shown to be essential to bring the NoRC complex to the rDNA promoters in order to make epigenetic modifications and form heterochromatin, and to bring the rDNA promoters to the nucleolus (69–71). It has been shown that other RNAs with similar structures are specifically recognized by BAZ2A and lead to heterochromatin formation. This is the case of the TERRA RNA that targets the formation of heterochromatin at the telomeres (72). It is therefore possible that the NoRC associated RNA, the elusive MIR6724 and the NA_ETS-RNA derived transcripts shown here are all, in fact, produced in order to regulate the chromatin structure of rDNA copies, as well as their localization and transcription levels. There is, however, no high-throughput analysis of BAZ2A interactions available in the literature and so this remains speculative for now.

Together the results presented here indicate that the annotation of the human genome is far from complete and underscore the value of direct analysis of RNA-Seq alignment profiles. Clearly, the number of non-annotated genes is not limited to the 111 genes identified here since we used stringent filters on our TGIRT-seq datasets, non-exhaustive sequencing techniques and did not consider sporadically expressed low abundance RNA. Deeper sequencing in different tissues and cell lines, and relaxed filters will uncover many additional yet to be annotated genes. The annotation-independent analysis used in this study might also be of value for identifying biomarkers expressed only in diseased tissues. These types of biomarkers by definition would not be annotated in normal genomes.

## DATA AVAILABILITY

Breast, prostate and ovarian as well as DKC1 depleted and lipofectamine SKOV3ip1 fragmented ribodepleted TGIRT-Seq datasets were deposited to the NCBI Gene Expression Omnibus (GEO; https://www.ncbi.nlm.nih.gov/geo/) under the accession number GSE126797.

## Supplementary Material

gkaa028_Supplemental_Files
