# Supplementary material for: Reducing the structure bias of RNA-Seq reveals a large number of non-annotated non-coding RNA
Source: Nucleic Acids Res. 2020 Jan 25;48(5):2271–86. doi: 10.1093/nar/gkaa028 (PMC7049693; doi:10.1093/nar/gkaa028)
Supplement: gkaa028_Supplemental_Files [file gkaa028_supplemental_files.zip › Figures_supp_V2020_3.pdf]

Supplementary Material

Boivin et al., 2020

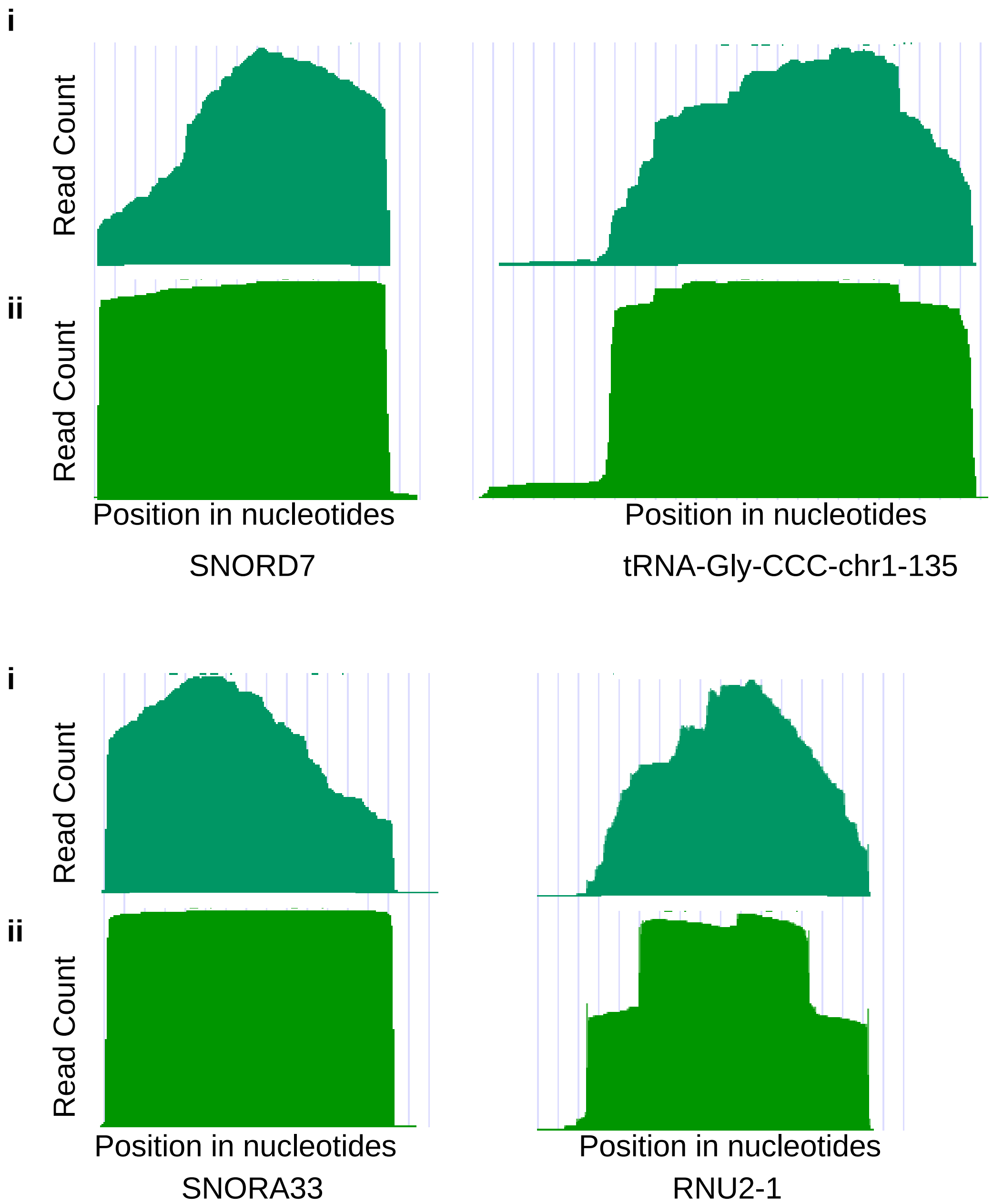

Figure S1. **Examples of read alignment profiles from fragmented and non-fragmented TGIRT-Seq datasets.** The read profiles of 4 different ncRNAs obtained using TGIRT-Seq of fragmented (i) and non-fragmented (ii) samples. The graph represents read alignment coverage (read count) per position.

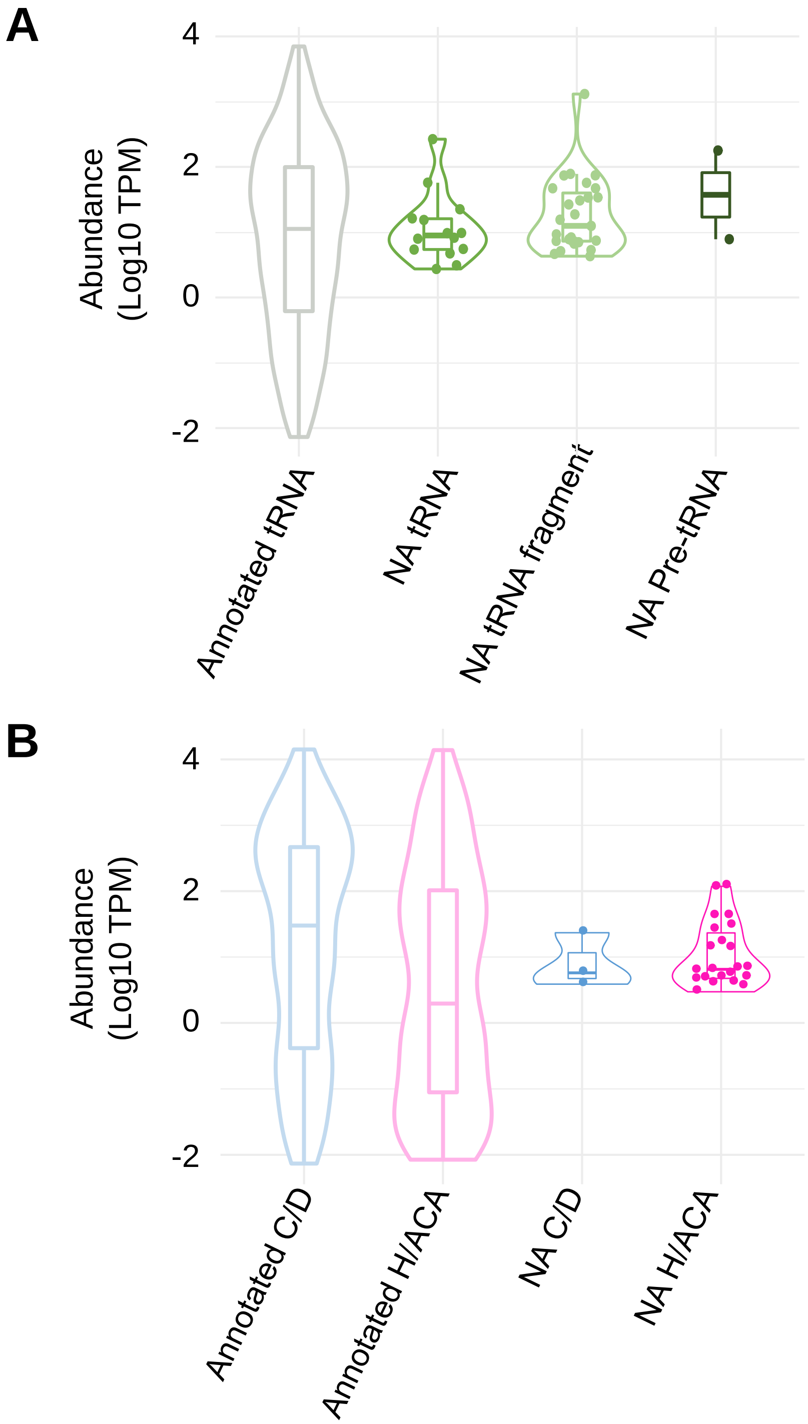

Figure S2 (related to Figure 2). NA_RNAs exhibit biotype specific expression patterns. (A) The abundance of annotated and non-annotated (NA) tRNAs, tRNA fragments and pre-tRNA was determined using non-fragmented SKOV3ip1 TGIRT-Seq as described in Figure 2. (B) The abundance of annotated and non-annotated (NA) C/D & H/ACA snoRNAs was determined as described in A. The average abundance in transcripts per million of the different RNA was determined from three replicates. Individual data points are not represented for annotated RNAs in (A) and (B) to reduce crowdedness.

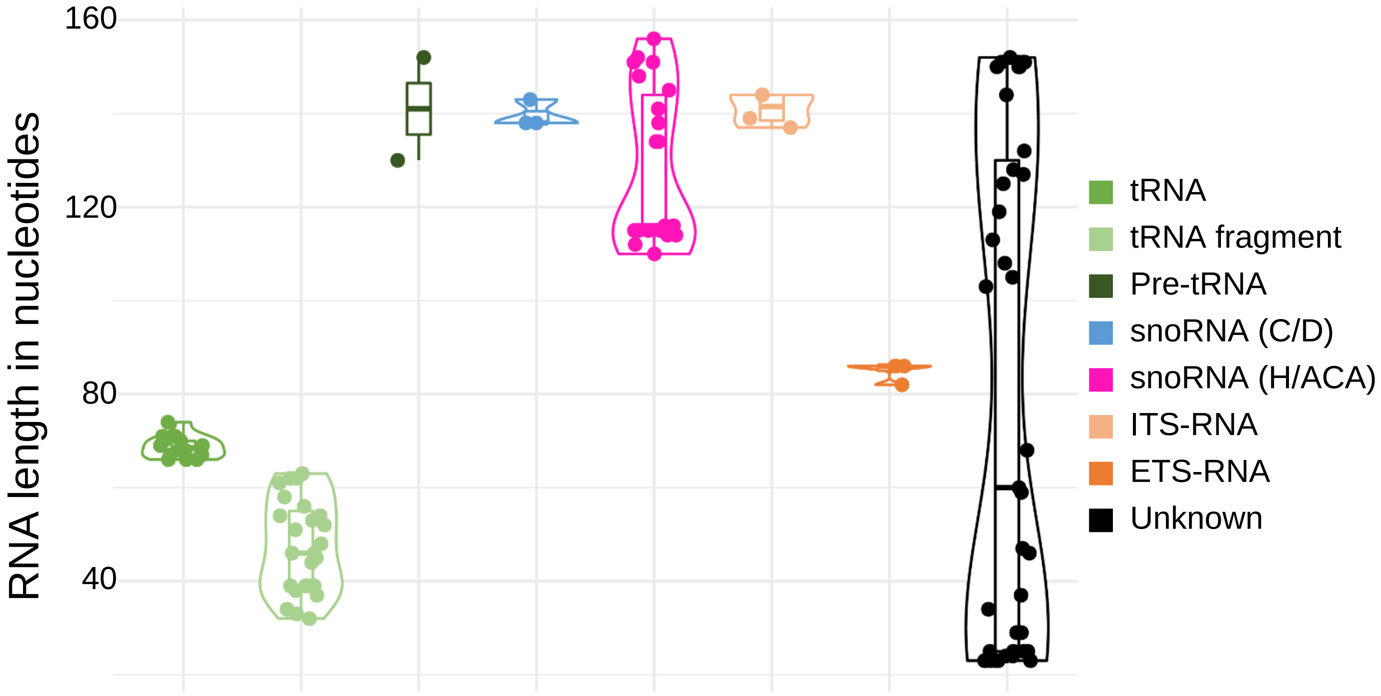

Figure S3 (related to Figure 1). Size distribution of NA_RNA. The length of the NA_RNA was determined as described in the methods section and shown by biotype in the form of a violin plot.

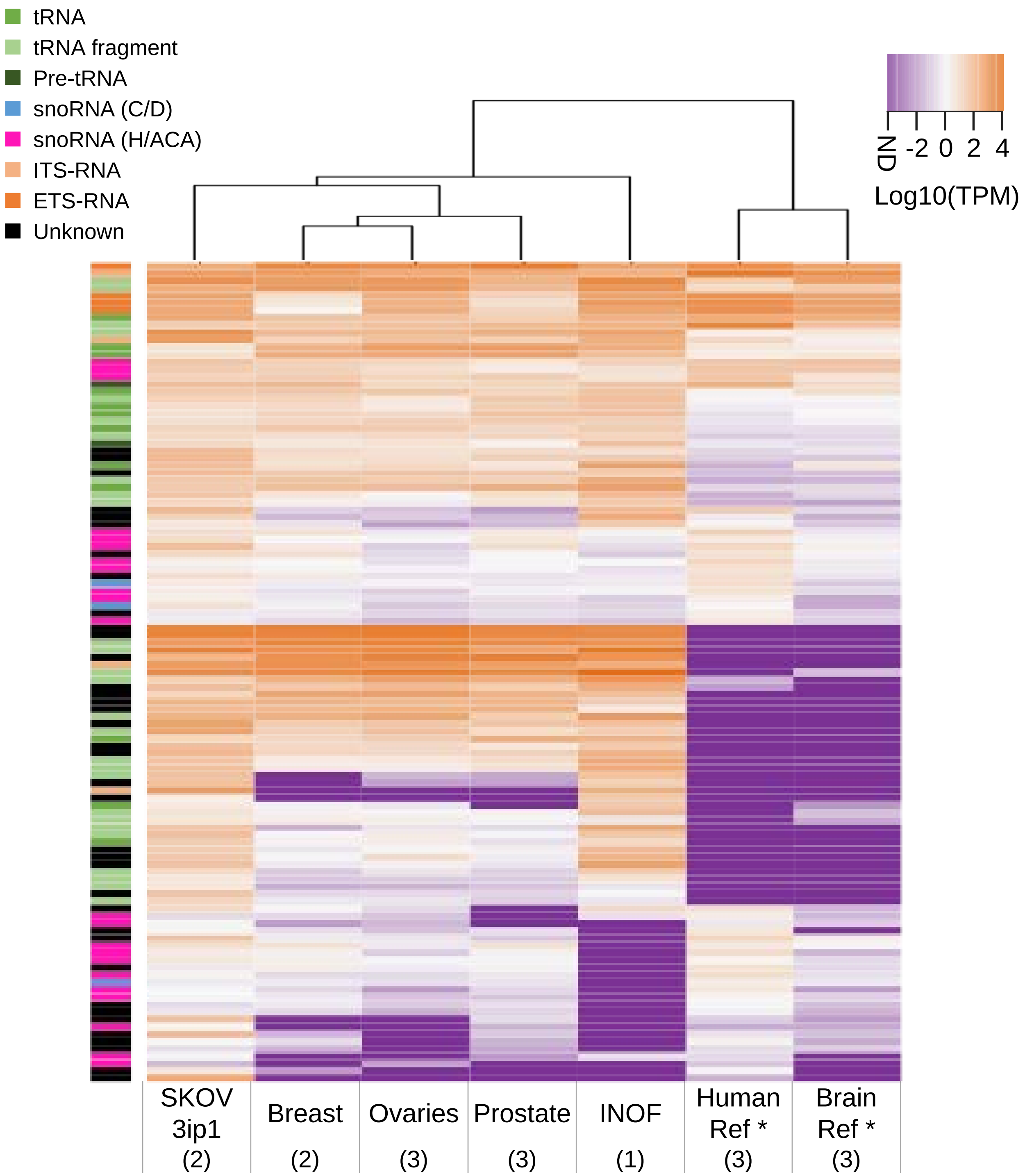

Figure S4. The NA_RNA expression is not restricted to a single tissue or cell line. The abundance of the different NA_RNA was determined in different tissues (breast, ovaries and prostate) as well as immortalized normal (INOF) and cancer cell lines (SKOV3iP1) using ribo-depleted, fragmented TGIRT-Seq. The human reference and brain reference data sets were obtained from (1). Biotypes are indicated on the left (legend on top left).

1- Nottingham,R.M., Wu,D.C., Qin,Y., Yao,J., Hunicke-Smith,S. and Lambowitz,A.M. (2016) RNA-seq of human reference RNA samples using a thermostable group II intron reverse transcriptase. RNA, 22, 597–613.

A

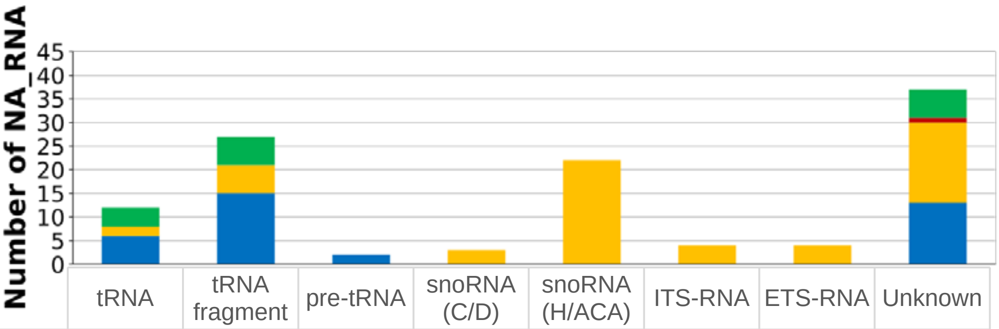

	tRNA	tRNA fragment	pre-tRNA	snoRNA (C/D)	snoRNA (H/ACA)	ITS-RNA	ETS-RNA	Unknown
antisense_intron	4	6	0	0	0	0	0	6
antisense_exon	0	0	0	0	0	0	0	1
intron	2	6	0	3	22	4*	4*	17
intergenic	6	15	2	0	0	0	0	13

B

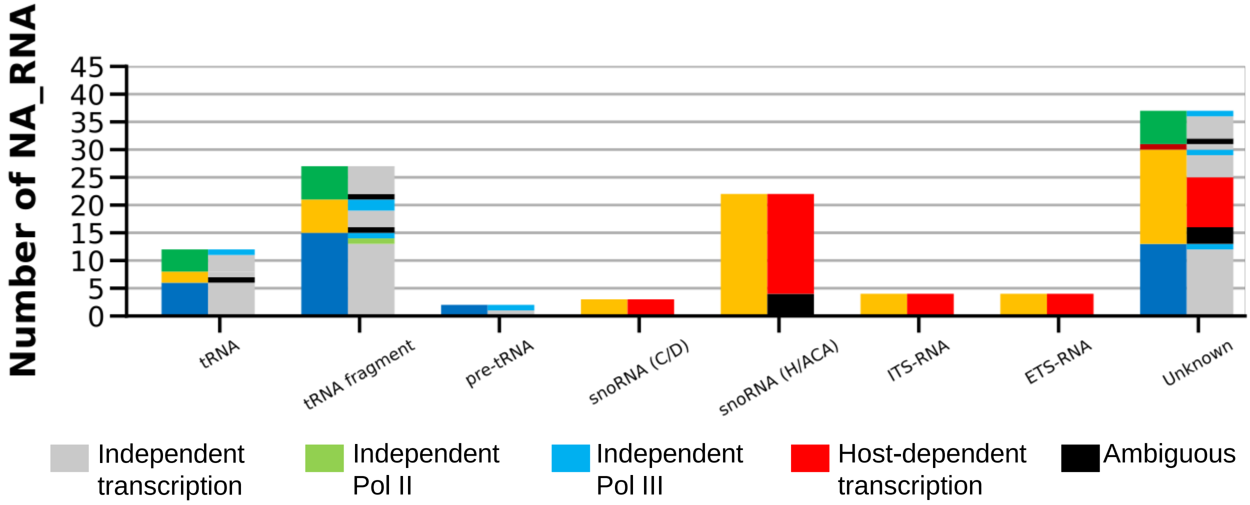

Figure S5. The NA_RNAs exhibit class specific genomic location and mechanism of gene expression. (A) The genomic location of the different NA_RNAs was determined for each biotype and the number of RNA of each biotype per location illustrated in the form of a histogram. The asterisks indicate RNA embedded in the 45S external transcribed spacers (ETS) or the internal transcribed spacer (ITS). (B) The expression mechanism of the NA_RNA by biotype is indicated in the form of a histogram. NA_RNA independently transcribed by RNAPII and RNAPIII were identified by the enrichment of RNAPII or RNAPIII ChIP-seq near the RNA 5' end. Host gene dependent NA_RNAs are identified as intronic RNA showing Pearson and Spearman correlation >0.2 with their host gene. Host independent NA_RNAs were identified as intronic RNA with a Pearson and Spearman correlation <-0.2 with the host gene. Intronic NA_RNAs / host correlation between -0.2 and 0.2 are considered ambiguous. For examples of NA_RNA / host gene correlation see Figure S6.

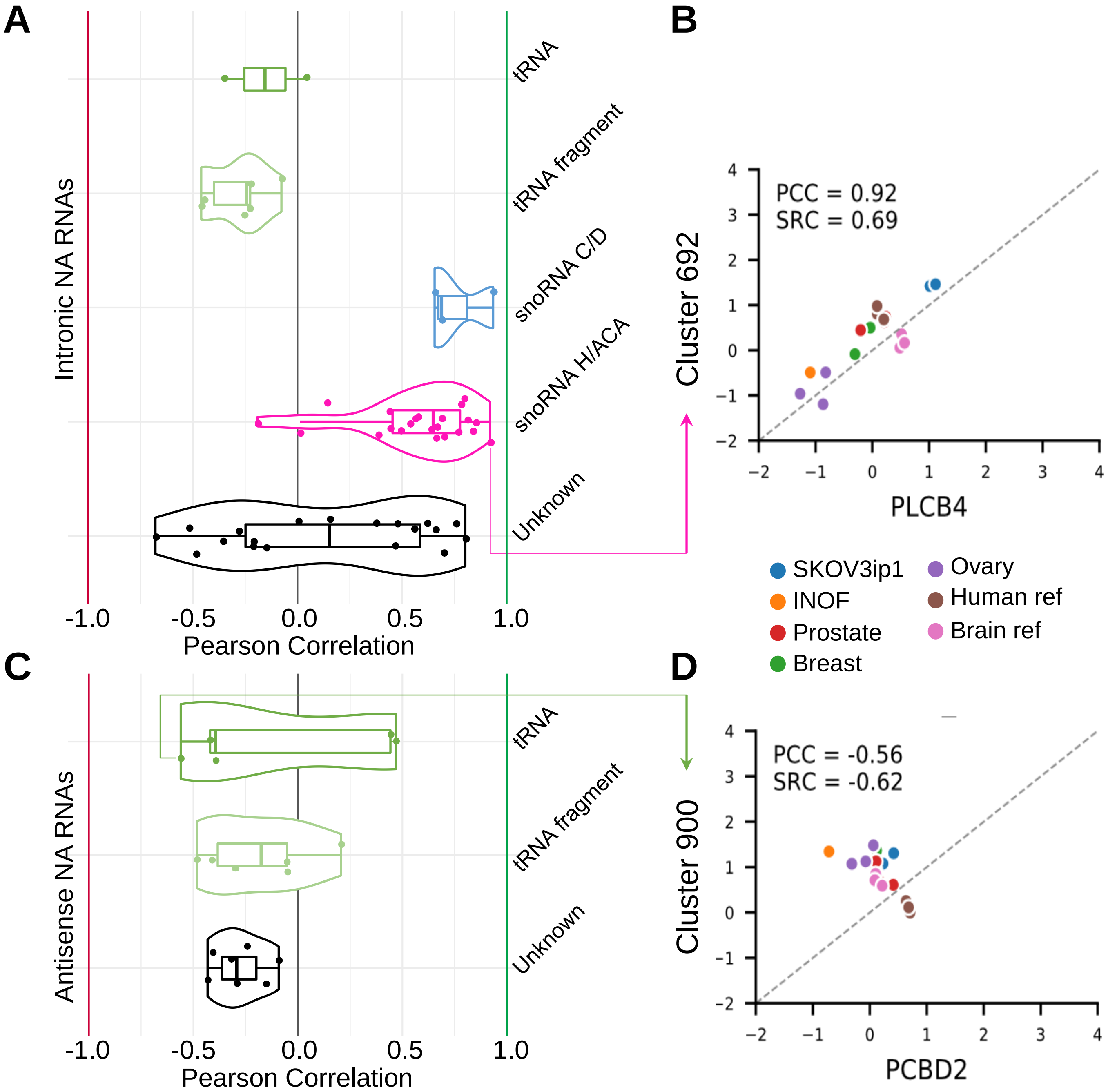

Figure S6. **The correlation between NA_RNA and their host gene expression depends on the NA_RNA transcription sense.** (A) Distribution of Pearson correlation of the intronic NA_RNAs and their host gene expression is shown by biotype. (B) Example of positively regulated NA_RNA/host gene expression. The expression of the intronic H/ACA snoRNA cluster 692 and its host gene in different tissues and cell lines is shown in the form of a scatter plot. The position of the snoRNA in its expression cluster shown in A is indicated by arrows. (C) Distribution of Pearson correlation of antisense NA_RNAs and their corresponding gene expression is shown by biotype. (D) Example of anti-correlated NA_RNA/host gene expression. The expression of antisense tRNA cluster 900 and its corresponding gene was determined as described in B. The sequencing data sets considered for the scatter plots are indicated with a color legend between panels B and D.

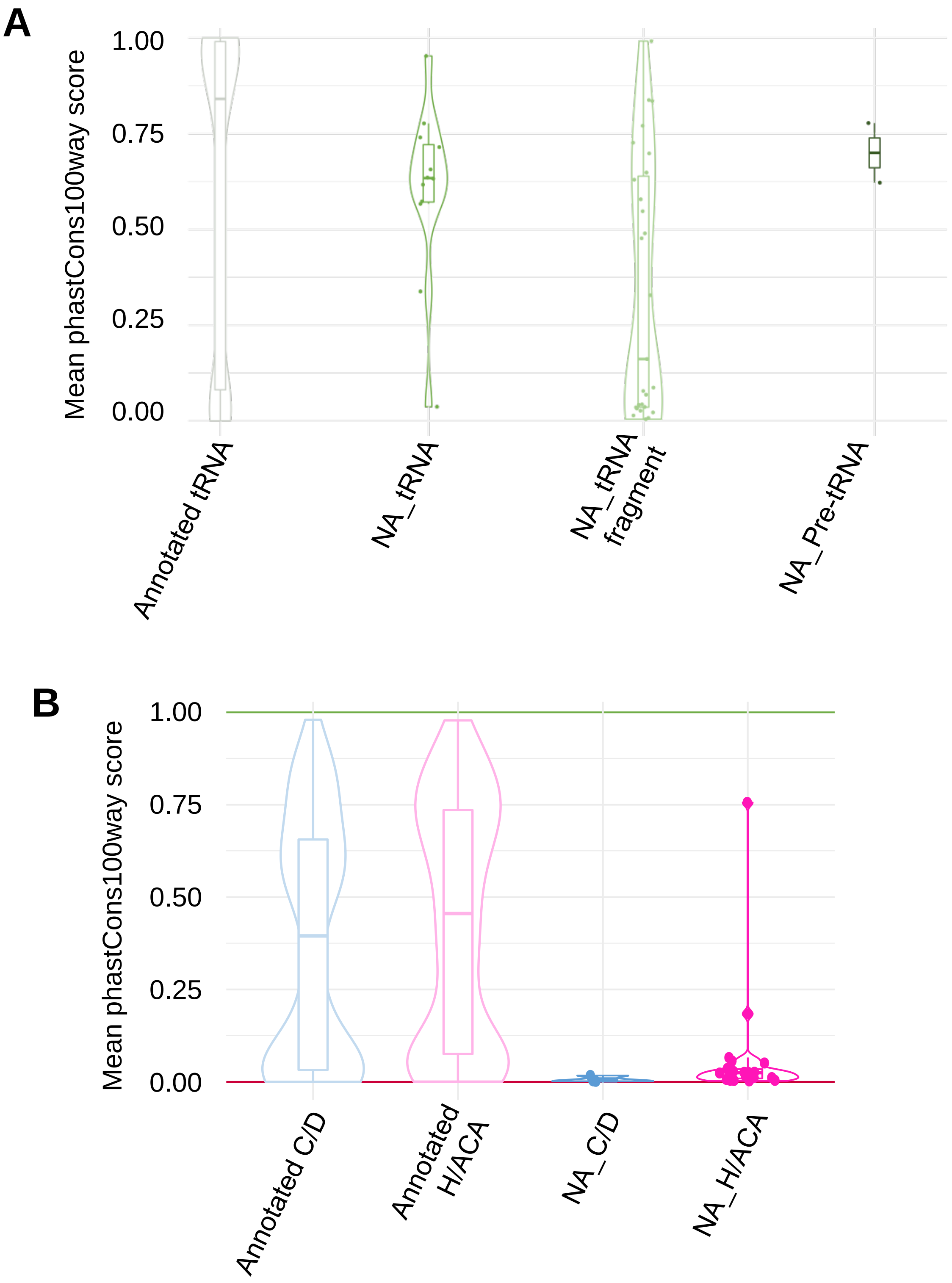

Figure S7 (related to Figure 3). NA_tRNAs are more conserved than their snoRNA counterparts. (A) Violin plot representing the distribution of the mean PhastCons100way score for annotated tRNAs, _NA_tRNAs, NA_tRNA fragments and NA_pre-tRNA. (B) Violin plot representing the distribution of the mean PhastCons100way score for annotated and NA C/D & H/ACA snoRNAs.

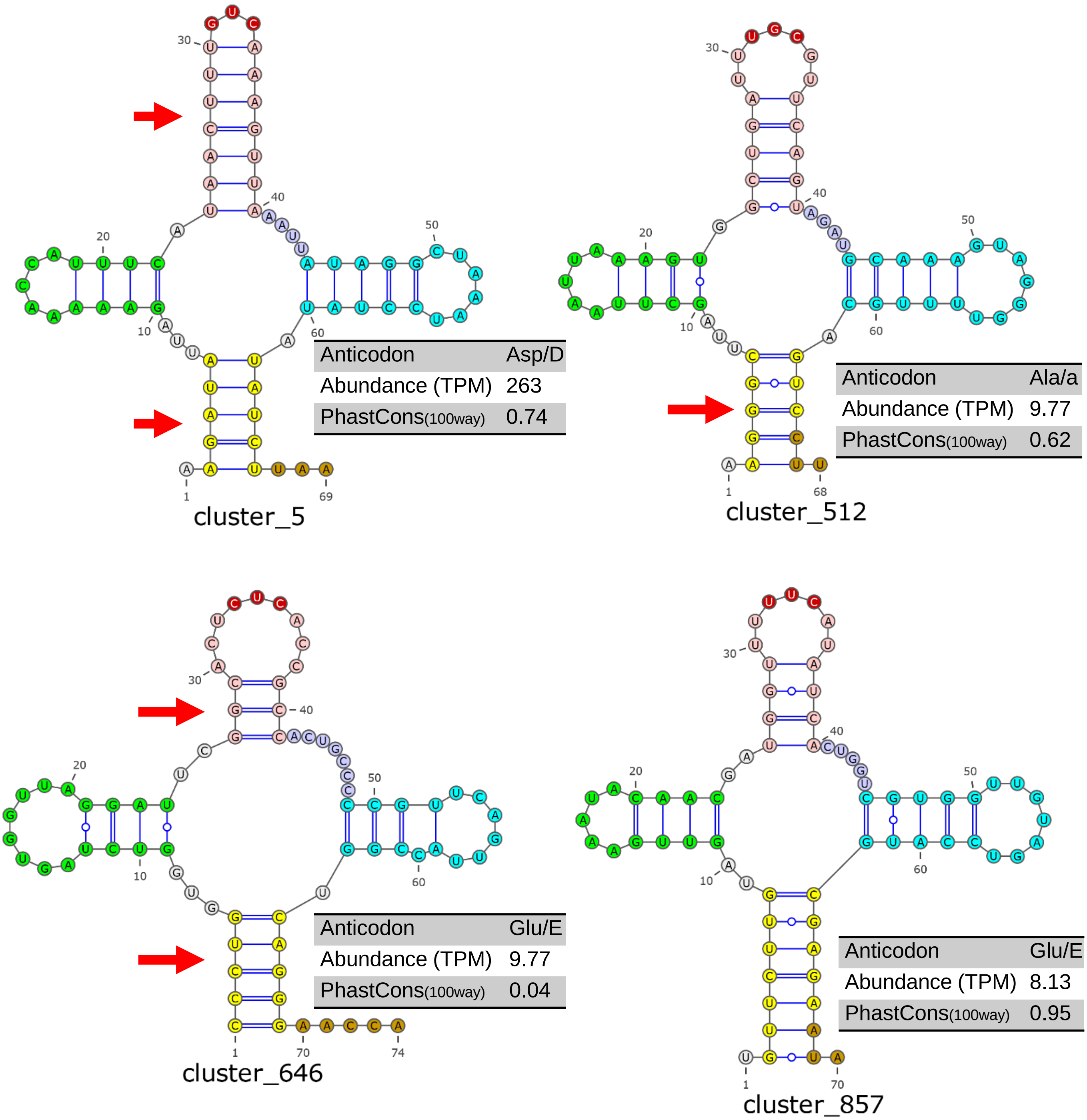

Figure S8. The majority of NA_tRNAs form imperfect tRNA structures. The secondary structure of the 6 NA_tRNAs were folded using turbofold and Information on the anticodon, expression level in non-fragmented SKOV3ip1 datasets (in TPM) and conservation (average phastCons100way conservation score) are indicated on the right. The acceptors stem, D arm, anitcodong arm and variable loop are indicated in yellow, green, red and magenta respectively. Deviation from the tRNA consensus are indicated by arrows.

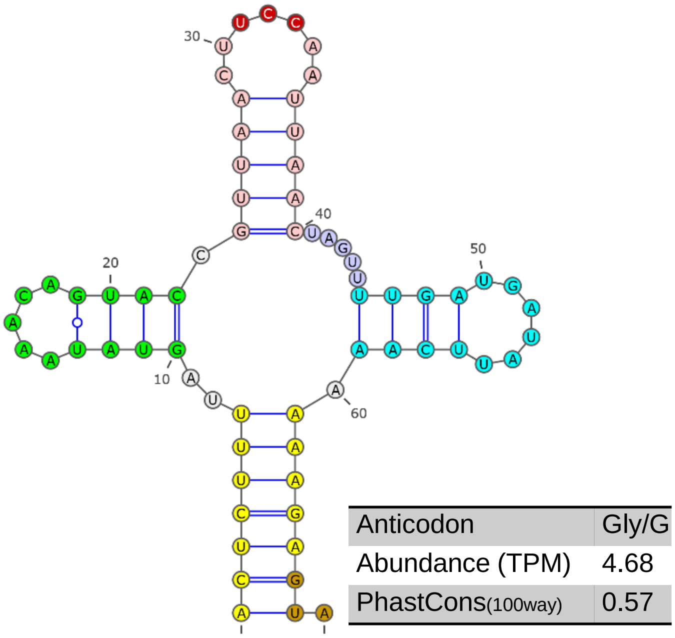

cluster_895

Anticodon	Gly/G
Abundance (TPM)	4.68
PhastCons(100way)	0.57

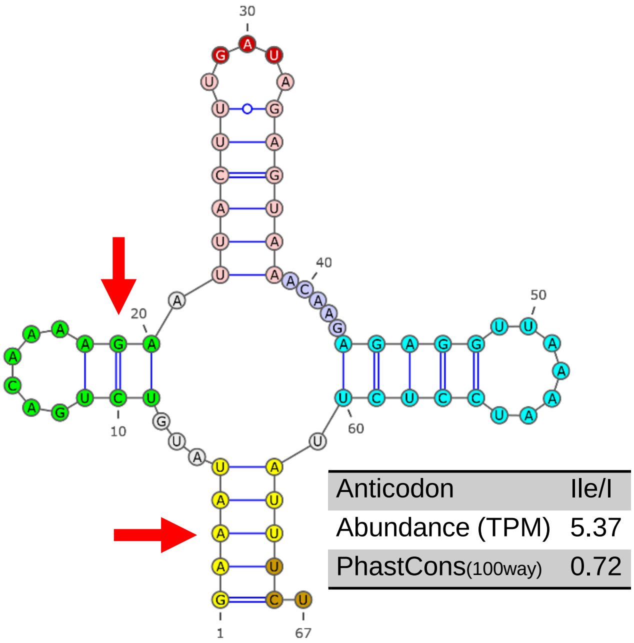

cluster_1133

Anticodon	Ile/I
Abundance (TPM)	5.37
PhastCons(100way)	0.72

Figure S8 (continued). The majority of NA_tRNAs form imperfect tRNA structures. The secondary structure of the 6 NA_tRNAs were folded using turbofold and Information on the anticodon, expression level in non-fragmented SKOV3ip1 datasets (in TPM) and conservation (average phastCons100way conservation score) are indicated on the right. The acceptors stem, D arm, anitcodong arm and variable loop are indicated in yellow, green, red and magenta respectively. Deviation from the tRNA consensus are indicated by arrows.

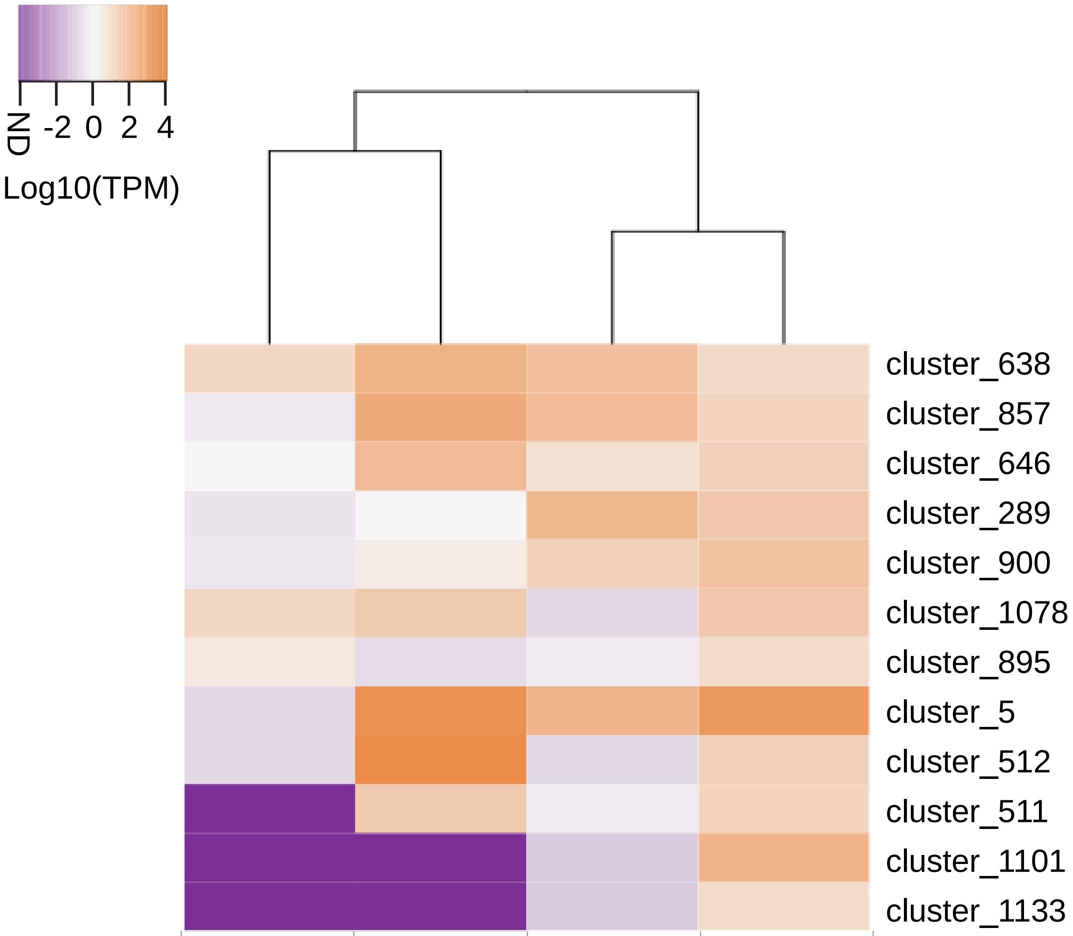

**Method**	YAMAT-Seq	Hydro-tRNAseq	DM-TGIRT-Seq	Non-fragmented TGIRT-Seq
**Cell-line**	MCF7	HEK293 (Flp-In T-Rex)	HEK293T	SKOV3ip1

Figure S9. The majority of the NA_tRNAs are detected in datasets using dedicated selective tRNA sequencing methodologies. The expression of the NA_tRNA was examined in sequencing datasets using tRNA specific library preparation techniques (Hydro-tRNAseq, YAMAT-Seq and DM-TGIRT-Seq). RNA abundance in log10 (TPM) is used to generate unsupervised clusters as indicated in Figure 3. The color legend indicates the abundance level in TPM (ND: not detected).

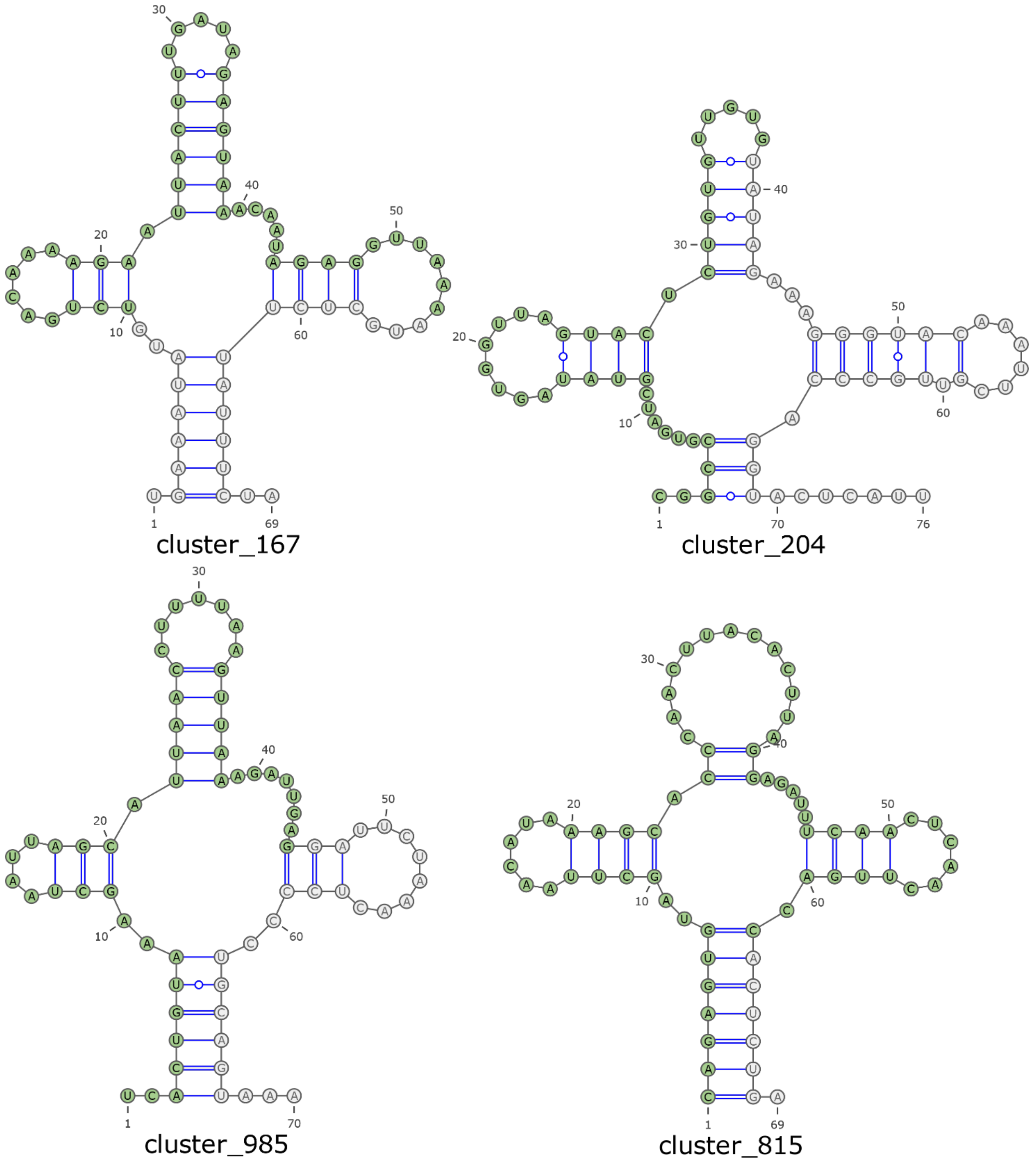

Figure S10 (related to Figure 5). The extension of the NA_tRNA fragments may fold into tRNA structures. The sequence surrounding the NA_tRNA fragments that constitutes the complete tRNA sequence was folded using Turbofold and the sequence corresponding to the detected fragments highlighted in green.

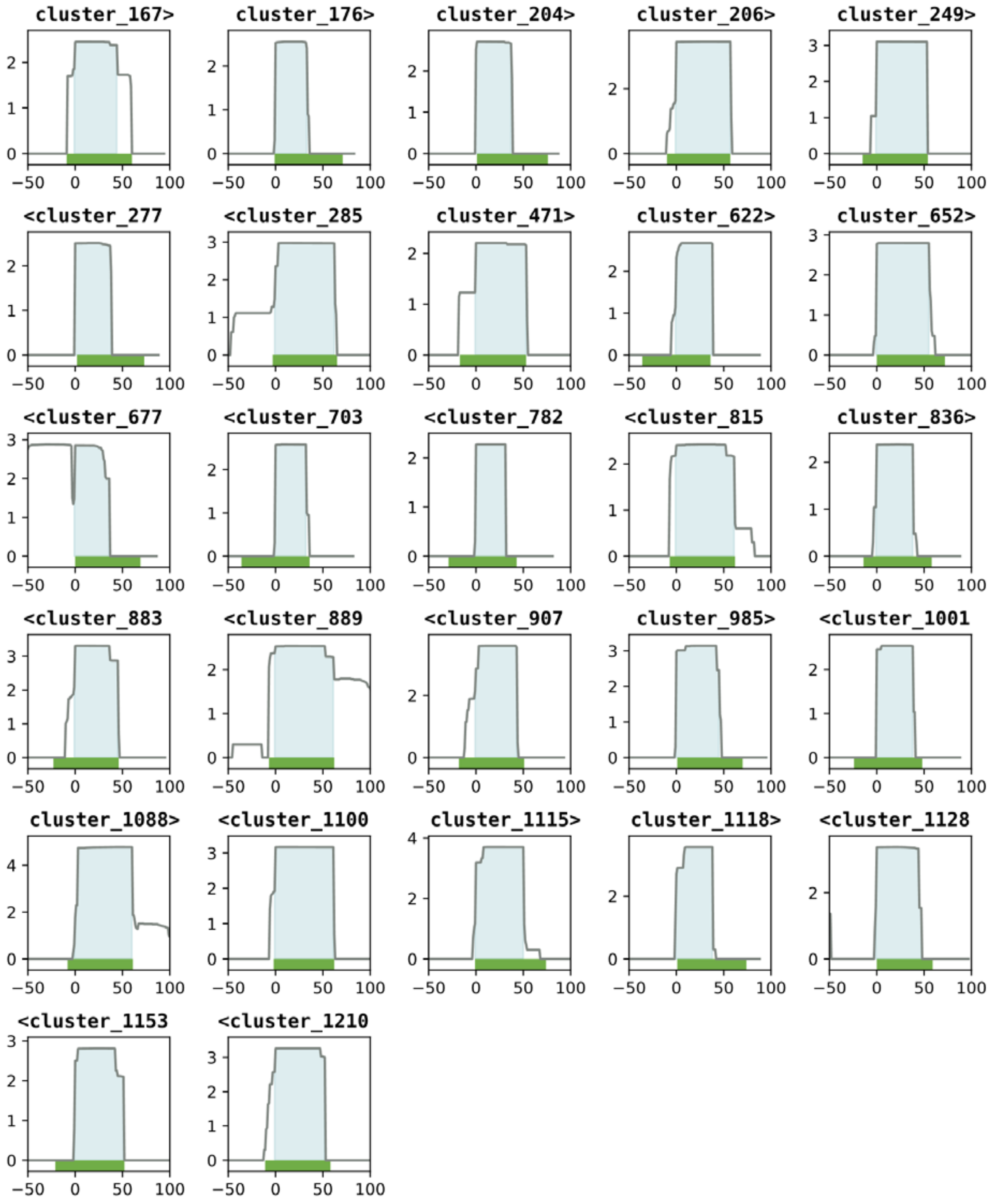

Figure S11 (related to Figure 5). Examples of the sequencing read distribution associated with the NA_tRNA fragments. Read accumulation patterns determined by non-fragmented SKOV3ip1 TGIRT RNA-Seq are shown relative to the loci of the NA_tRNA fragments. The X axes represent the genomic coordinates in relation to the cluster start and the Y axes are the log10 read counts. The region corresponding to the tRNA fragment is highlighted in blue and the full mature tRNA sequence inferred from the reference tRNA sequence illustrated in the form of green boxes. The arrow heads indicate the transcription sense.

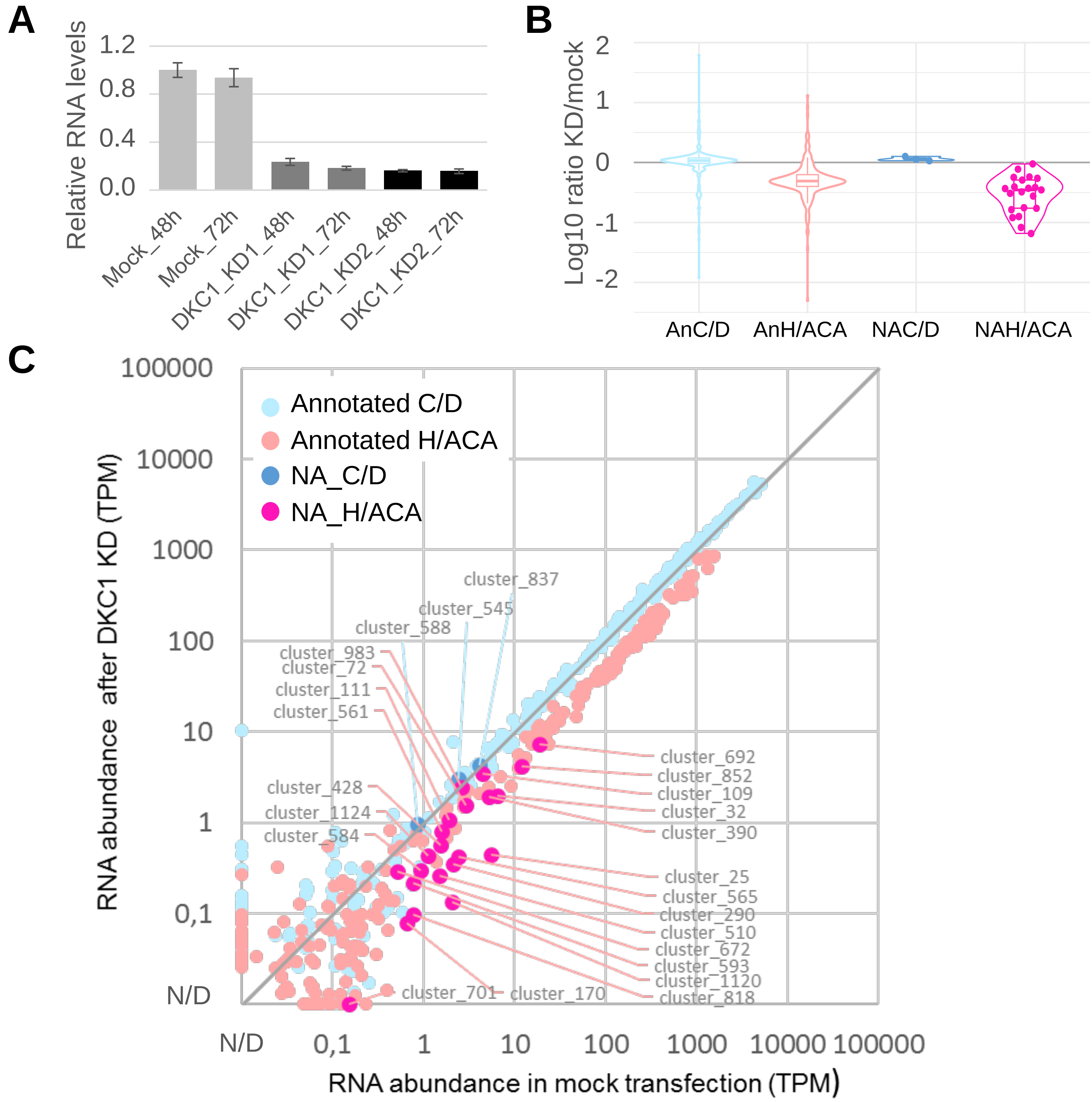

Figure S12 (related to Figure 6). Depletion of the H/ACA snoRNA binding protein Dyskerin (DKC1) inhibits the accumulation of the NA_H/ACA snoRNAs. (A) The abundance of DKC1 mRNA as determined by qRT-PCR (in triplicates) in mock transfection or after transfection of two independent siRNAs (KD1 and KD2) targeting DKC1 sequence. (B) The impact of DKC1 knockdown on the abundance of known and NA_snoRNAs is shown in the form of a violin plot. AnC/D, AnH/ACA, NA_C/D and NA_H/ACA indicate annotated (An) and NA C/D and HACA snoRNA. (C) The global impact of the DKC1 on the C/D and H/ACA snoRNA abundance is shown in the form of a scatter plot. The different types of snoRNAs are indicated on the top left.

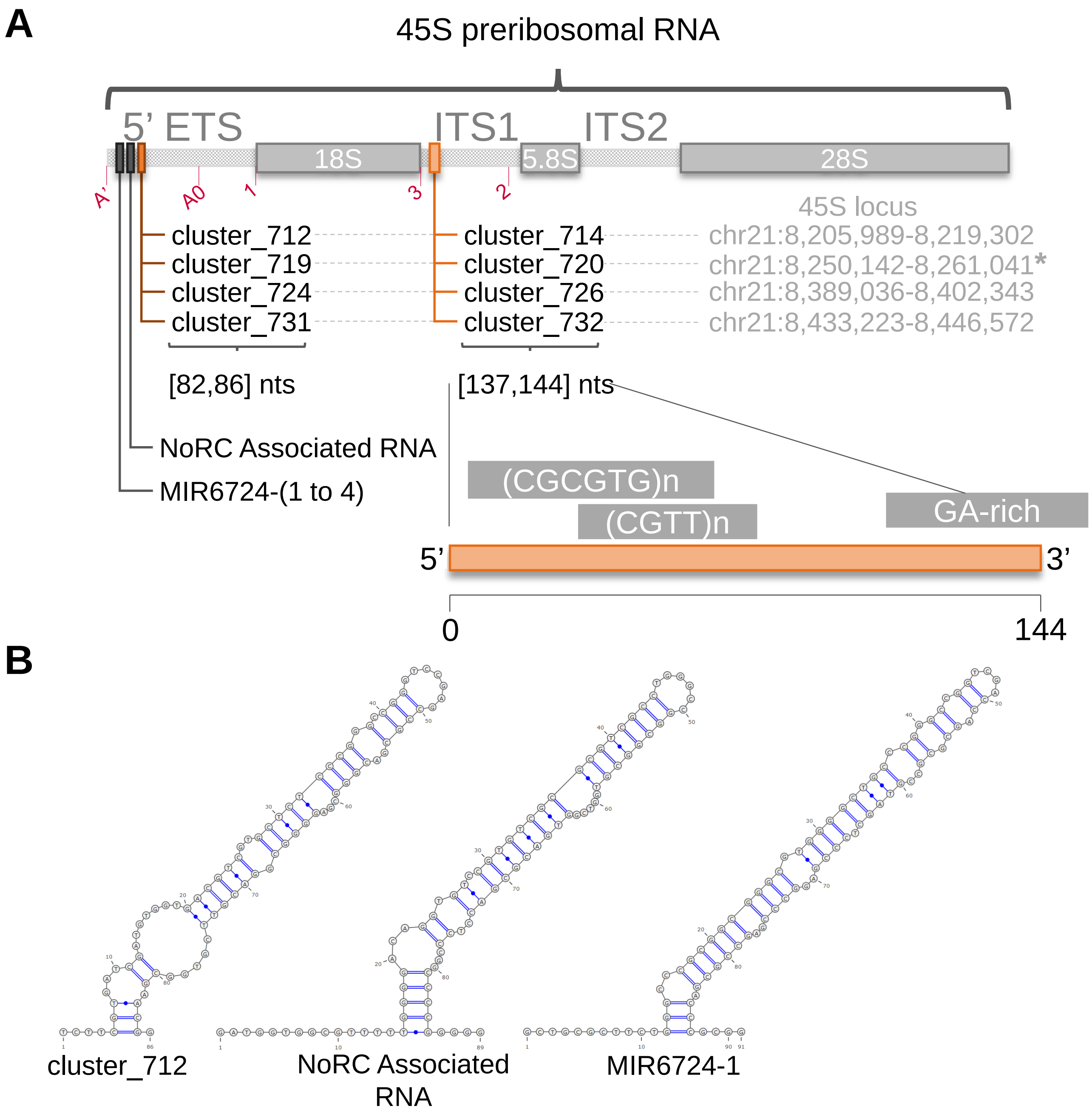

Figure S13. Genomic location and structure of NA_RNAs embedded in 45S preribosomal RNA spacer sequence. (A) Diagram showing the position of NA_RNAs within the rDNA sequence repeat. Boxes indicate the 18S, 5.8S and 28S mature rRNA sequence. Lines indicate the external (ETS) and internal (ITS) transcribed spacers. The position of the previously established processing sites (1) is indicated in red. The name and coordinates of the NA_RNA is indicated below the rDNA repeat. The position of the short tandem repeats found within the ITS-RNA sequence is shown at the bottom. The asterisk indicates approximate position of non-anotated 45S locus. (B) The structure of cluster 712 was predicted using Turbofold and compared to the structure of known pRNA and MIR6724-1 precursor.

1- Mullineux,S.-T. and Lafontaine,D.L.J. (2012) Mapping the cleavage sites on mammalian pre-rRNAs: where do we stand? Biochimie, 94, 1521–1532.

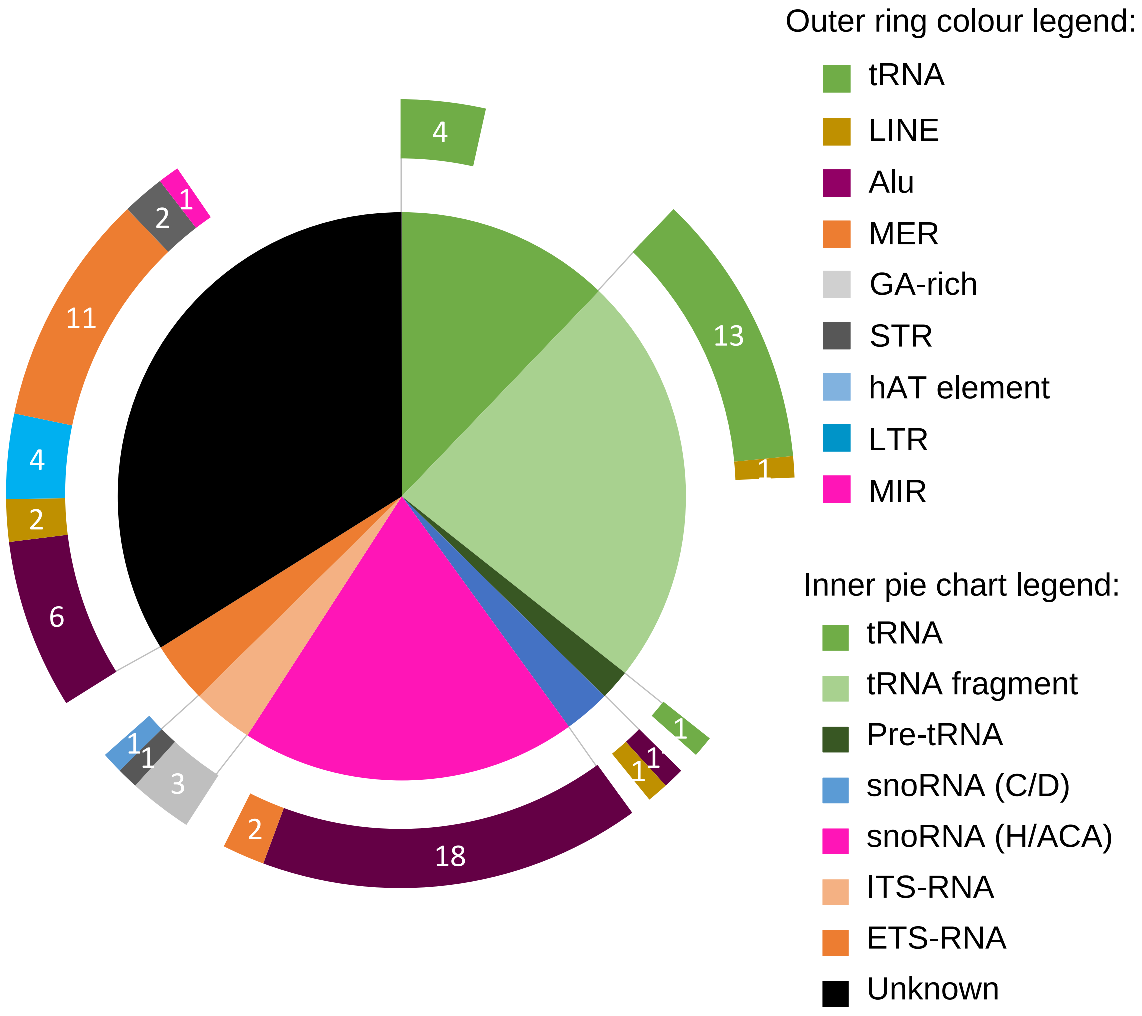

Figure S14. The majority of the NA_RNA are located within genomic sequence repeats. The different NA_RNA biotypes are illustrated in the form of a pie chart as indicated in Figure 1F and their overlapping repeated elements identified by Repeatmasker are illustrated in the outer ring.

Table S1. RNA-seq dataset accessions and descriptions.

Sequencing dataset ID	Source database	Description
GSM2631743	GEO*	SKOV3ip1 non-fragmented ribodepleted TGIRT-Seq RNA
GSM2631744		
GSM2631741	GEO	SKOV3ip1 fragmented ribodepleted TGIRT-Seq RNA
GSM2631742		
GSM2631745	GEO	SKOV3ip1 fragmented ribodepleted viral RNA-Seq RNA
GSM2631746		
GSM2997959	GEO	INOF fragmented ribodepleted TGIRT-Seq RNA
SRR2912443	SRA**	Human reference ribodepleted fragmented TGIRT-Seq RNA
SRR2912444		
SRR2912446		
SRR2912479	SRA	Brain reference ribodepleted fragmented TGIRT-Seq RNA
SRR2912481		
SRR2912483		
GSM2521595	GEO	Hydro-tRNAseq RNA
GSM2521596		
GSM2521597		
GSM2521598		
SRR5168440	SRA	YAMAT-Seq RNA
SRR5168441		
SRR5168442		
GSM1624818	GEO	Demethylase TGIRT-Seq RNA
GSM1624819		

*: NCBI Gene Expression Omnibus (GEO)
**: NCBI Short Read Archive (SRA)

Table S2. RNA-Seq data processing tools and parameters.

Tool name	Parameters
Cutadapt	--minimum-length 2 --front GATCGTCGGACTGTAGAACTCTGAACGTGTAGATCTCGGTGGTCGCCGTATCATT --adapter AGATCGGAAGAGCACACGTCTGAACTCCAGTCACATCACGATCTCGTATGCCGTCTTCTGCTTG -G GATCGTCGGACTGTAGAACTCTGAACGTGTAGATCTCGGTGGTCGCCGTATCATT -A AGATCGGAAGAGCACACGTCTGAACTCCAGTCACATCACGATCTCGTATGCCGTCTTCTGCTTG --match-read-wildcards --quality-cutoff 3 --paired-output
Trimmomatic	PE –phred33 TRAILING:30
STAR	--runMode genomeGenerate –sjdbOverhang 99
	--runeMode alignReads –readFilesCommand zcat –outReadsUnmapped Fastx –outFilterType BySJout –outStd Log –outSAMunmapped None –outSAMtype BAM SortedByCoordinate –outSAMprimaryFlag AllBestScore –alignIntronMax 1250000
Bowtie	bowtie2-build
	bowtie2 –local -q --minins 13 –1 STAR_aligned/Unmapped.out.mate1 -2 STAR_aligned/Unmapped.out.mate2 -S
samtools	samtools view –bS bowtie2/aligned.sam > bowtie2/aligned.bam
	samtools merge aligned_allmerged.out.bam STAR_aligned/aligned.sortedByCoord.out.bam bowtie2/aligned.bam

Table S3. Turbofold II predictor reference gene sets

Biotype	RNAcentral ID set 1	RNAcentral ID set 2
tRNA	URS00006F85D5	URS0000172BA5
	URS00006E0B11	URS000032B149
	URS00002F2AEC	URS000052A090
	URS000014D40F	URS000039A306
	URS00006F85D5	URS000052A090
	URS00002DDD59	URS00004CF38D
	URS00006F85D5	URS00001232F5
	URS0000225EE1	URS00005386AB
	URS00006F85D5	URS000052A090
	URS00001232F5	URS000064F320
H/ACA snoRNA	URS00006FC667	URS000063BFC1
	URS00006A1082	URS00006C38E6
	URS0000701BA1	URS0000724753
	URS0000634EB5	URS0000704142
	URS000064930D	URS00001BD443
	URS0000718C0A	URS0000723018
	URS00006F3B25	URS0000056589
	URS000062AC4B	URS000064B135
	URS00008139D1	URS0000285CCB
	URS000019135F	URS00006EDADE

Table S4. NA_snoRNA characteristics.

cluster id	snoatlas id	predicted type	snoscan target	snoGPS target	repeated element overlap
**cluster_545**		C/D	18S-Gm601		x
**cluster_588**		C/D	18S-Am468		x
**cluster_837**		C/D	28S-Am1503		x
**cluster_25**		H/ACA		18S-U822	AluSz6
**cluster_32**	snoID_0757	H/ACA			AluJo
**cluster_72**	snoID_0766	H/ACA		18S-U34	AluJr
**cluster_109**	snoID_0749	H/ACA		28S-U3727	AluJr
**cluster_111**		H/ACA			AluJo
**cluster_170**		H/ACA			AluSc8
**cluster_290**		H/ACA		18S-U1625	MER58A
**cluster_390**	snoID_0759	H/ACA			AluJb
**cluster_428**	snoID_0784	H/ACA			AluY
**cluster_510**	snoID_0769	H/ACA			AluSq2
**cluster_561**		H/ACA		18S-U110	MER20
**cluster_565**	snoID_0801	H/ACA			AluSx1
**cluster_584**	snoID_0787	H/ACA			AluY
**cluster_593**	snoID_0855	H/ACA			AluY
**cluster_672**		H/ACA		18S-U801	x
**cluster_692**	snoID_0826	H/ACA			AluJb
**cluster_701**	snoID_0750	H/ACA		28S-U1656	AluY
**cluster_818**		H/ACA			AluSz
**cluster_852**	snoID_0671	H/ACA			x
**cluster_983**		H/ACA			AluJb
**cluster_1120**	snoID_0785	H/ACA			AluSz
**cluster_1124**	snoID_0804	H/ACA			AluY
