# Supplementary material for: Reducing the structure bias of RNA-Seq reveals a large number of non-annotated non-coding RNA
Source: Nucleic Acids Res. 2020 Jan 25;48(5):2271–86. doi: 10.1093/nar/gkaa028 (PMC7049693; doi:10.1093/nar/gkaa028)
Supplement: gkaa028_Supplemental_Files [file gkaa028_supplemental_files.zip › SUPPLEMENTARY_FILE_2.pdf]

Supplementary file 2: tRNA fragment extension alignment file

###For all alignment entries, the NAR sequence comes first, then the extended NAR sequence, then the
###most similar tRNA found in RNAcentral (v10) (RNAcentral ID is given).

```
cluster_1001_frag           GUGUAAUGAUUAGCACUCUGGACUUUGAAUCCAGGAUCC
cluster_1001_ext   GACCUCUUGGUGUAAUGAUUAGCACUCUGGACUUUGAAUCCAGGAUCC--------------ACUGGGACCU
URS000070F1D2      GGUCCCAUGGUGUAAUGGUUAGCACUCUGGACUUUGAAUCCAGCAAUCCGAGUUCGAAUCUCGGUGGGACCU
                   *  * * ********** ************************* *  *                ********


cluster_1088_frag          AGGUUAACAUAAAGCACCCAACUUACACUUAGGAGAUUUCAACUUAACUUGACCGCUCUGA
cluster_1088_ext   AAUAAAGGAGGUUAACAUAAAGCACCCAACUUACACUUAGGAGAUUUCAACUUAACUUGACCGCUCUGA
URS00001232F5      CAGAGUGUAGCUUAACAUAAAGCACCCAACUUACACUUAGGAGAUUUCAACUUAACUUGACCGCUCUGA
                    * *  * ** **********************************************************


cluster_1100_frag  AGUAAGGUCAGCUAAAUAAGCUAUCUGGCCCAUACCCCAAAAAUGUUGGUUAUACCCUUCCC
cluster_1100_ext   AGUAAGGUCAGCUAAAUAAGCUAUCUGGCCCAUACCCCAAAAAUGUUGGUUAUACCCUUCCCAU
URS000040608E      AGUAAGGUCAGCUAAAUAAGCUAUCGGGCCCAUACCCCGAAAAUGUUGGUUAUACCCUUCCCGU
                   ************************* ************ *********************** *


cluster_1115_frag   UUAAGAUGGCAGAGCCCAGUUAUUGCAUAAAACUUAAAACUUUACAAUCAG
cluster_1115_ext   AUUAAGAUGGCAGAGCCCAGUUAUUGCAUAAAACUUAAAACUUUACAAUCAG---CUCAACUCC--UUCUUAACAGCAUG
URS0000689D74      GUUAAGAUGGCAGAGCCCAGCAAUUGCAUAAAACUUAAAACUUUACAAUCAGAGGUUCAACUCCUCUUCUUAACA-----
                    *******************  ******************************     ********   *********


cluster_1118_frag  UGGCUCUGUUGCGCAAUGGAUAGCGCAUUGGACUUCUAA
cluster_1118_ext   UGGCUCUGUUGCGCAAUGGAUAGCGCAUUGGACUUCUAAAUUGCCCAGAUAUU-----UUCUAAUCUC--CAUUUUUUAUG
URS0000225EE1      -GGCUCUGUGGCGCAAUGGAUAGCGCAUUGGACUUCUAAUUC----AAAGGUUGUGGGUUCGAGUCCCACCAGAGUCG---
                    ******** ***************************** *      * *  **      *** * ** *  **    *


cluster_1128_frag             CAGGAACUGCUAACUCAUGCCCCCAUGUCUAACAACAUGGCUUUCUCA
cluster_1128_ext   GAGAAUGUAUGCAGGAACUGCUAACUCAUGCCCCCAUGUCUAACAACAUGGCUUUCUCA
URS0000027F06      GAGAAAGCUCGCAAGAACUGCUAACUCAUGCCCCCAUGUCUAACAACAUGGCUUUCUCA
                   ***** *   *** *********************************************


cluster_1153_frag  GGGCCAGUGGCGCAAUGGAUAACGCAUCUGACUACGGAUCAGAAGAUUCUAG
cluster_1153_ext   GGGCCAGUGGCGCAAUGGAUAACGCAUCUGACUACGGAUCAGAAGAUUCUAGAAAUGUGAGGCCAUGAGAUUU--
URS00002DDD59      GGGCCAGUGGCGCAAUGGAUAACGCGUCUGACUACGGAUCAGAAGAUUCUAGGUUC--GACUCCUGGCUGGCUCG
                   ************************* **************************      **  **  *     *


cluster_1210_frag        UGUUCUUGAAGUACAACGAUGGUUUUUCAUAUCAUUAGUCAUGGUUGUAGUCC
cluster_1210_ext   AUUGGUGUUCUUGAAGUACAACGAUGGUUUUUCAUAUCAUUAGUCAUGGUUGUAGUCCAUGUGAUAGAU
URS000025D745      GUUCUUGUAGUUGAAGUACAACGAUGGUUUUUCAUAUCAUUGGUCGUGGUUGUAGUCCGUGCGAGAAUA
                    **  ***  ******************************* *** ************ ** ** *


cluster_167_frag            UCUGACAAAAGAAUUACUUUGAUAGAGUAAACAAUAGAGGUUAAA
cluster_167_ext    UGAAAUAUGUCUGACAAAAGAAUUACUUUGAUAGAGUAAACAAUAGAGGUUAAAAUGCUCUUAUUUCUA
URS00006F85D5      GGAAAUAUGUCUGACAAAAGAACUACUUUGAUAGAGUAAACAACAGAGGUUAAAAUCCUCUUAUUUCUA
                    ********************* ******************** ************ ************


cluster_176_frag    GGGGUAUAGCUCAGUGGCAGAGCAUUUGACUGCA
cluster_176_ext    AGGGGUAUAGCUCAGUGGCAGAGCAUUUGACUGCAGAAUUAG---CCUAUAGCUGAAAUUAUAAUCAAAACUGUG--------
URS000014D40F      GGGGGUAUAGCUCAGUGGUAGAGCAUUUGACUGCAGAUCAAGAGGUCCCUGGUU-----------CAAAUCCGGGUGCCCCCU
                    **************** *****************   **     *  * * *           **** * * *


cluster_204_frag   CGGCCGUGAUCGUAUAGUGGUUAGUACUCUGUGUUGUG
cluster_204_ext    CGGCCGUGAUCGUAUAGUGGUUAGUACUCUGUGUUGUGUAUA--GAAA---GGGUACAAAUUCGUUGCCCAGGUACUCAUU
URS00002F2AEC      -GGCCGUGAUCGUAUAGUGGUUAGUACUCUGCGUUGUGGCCGCAGCAACCUCGGUUCGAAUCCGAGUCAC-GGCACC-A--
                    ****************************** ******      * **    *** * *** **    * * ** **  *


cluster_206_frag             U-----GAAAUACAACGAUGGUUUUUCAUAUCAUUGGUCGUGGUUGUAGCCCGUGCGAGAAUA
```

```
cluster_206_ext    UUCGUUGUUCU-----GAAAUACAACGAUGGUUUUUCAUAUCAUUGGUCGUGGUUGUAGCCC--GUGCGAGAAUA
URS000012E7F0      -----UAUUCUCGCACGGGCUACAACCACGACCAAUGAUAUGAAAAACCAUCGUUGUAUUUCAACUACAAGAAC-
                        * ****     *   ****** * *     * **** *     * * ******   *   * * ****



cluster_249_frag                  AAAAGAAUUACUUUGAUAGAGUAAACAAUAGAGGUUAAAAUCCUCUUAUUUCUA
cluster_249_ext    AGCAAUACAUCUCACAAAAGAAUUACUUUGAUAGAGUAAACAAUAGAGGUUAAAAUCCUCUUAUUUCUA
URS00006F85D5      GGAAAUAUGUCUGACAAAAGAACUACUUUGAUAGAGUAAACAACAGAGGUUAAAAUCCUCUUAUUUCUA
                    * ****  *** ********* ******************** *************************

cluster_277_frag                            UAUCAAGAGGUCCCCGGUUCAUAUCCAAGUGCCCUCUCC
cluster_277        CACAAUAUAAUUAAUGAAUUAA-GAUAUCAUUCUCUAUCAAGAGGUCCCCGGUUCAUAUCCAAGUGCCCUCUCC
URS00001F47B5      GGGGGUAUAGCUCAGGGGUAGAGCACUUGACUGCAGAUCAAGAGGUCCCUGGUUCAAAUCCAGGUGCCCCCU--
                        ****  * * *  *  *  *  * * *    ************* ****** ***** ****** **

cluster_285_frag     UAAGGUCGGCUAAAUAAGCUAUCGAGCCCAUACCCCGAAAAUGUUGGUUAUAUCCUUCCCAUA
cluster_285_ext    AAUAAGGUCGGCUAAAUAAGCUAUCGAGCCCAUACCCCGAAAAUGUUGGUUAUAUCCUUCCCAUAAGC
URS00002116D6      AGUAAGGUCAGCUAAAUAAGCUAUCGGGCCCAUACCCCGAAAAUGUUGGUUAUAUCCUUCCCGUACUA
                   * ******* **************** *********************************** **

cluster_471_frag                    AGCAUUAACCUUUUAAGUUAAAGACUAAGAGAAUCGCUAUCUCUUUACAGUGAC
cluster_471_ext    CACUGUAGAGCUGACCCAGCAUUAACCUUUUAAGUUAAAGACUAAGAGAAUCGCUAUCUCUUUACAGUGAC
URS0000206F1C      CACUGUAAAGCUAACUUAGCAUUAACCUUUUAAGUUAAAGAUUAAGAGAACCGACACCUCUUUACAGUGA-
                   ******* **** **  ************************ ******** **  * *************

cluster_622_frag                                          AUCUGAGGGUCCAGGGUUCAUAUCCCUGUUCAGGCACCA
cluster_622_ext    -UUUGGACAG--CAGCUGGUCACGACCCUCAGGCUUUCAAUCUGAGGGUCCAGGGUUCAUAUCCCUGUUCAGGCACCA
URS00006587E4      GCCUGGAUAGCUCAGUUGGU--AGAGCAUCAGACUUUUAAUCUGAGGGUCCAGGGUUCAAGUCCCUGUUCAGGCA---
                      **** **  *** ****   ** * **** **** *********************  **************

cluster_652_frag   GCCGUGAUCGUAUAGUGGUUAGUACUCUGUGCUGUGGCUGCAGCAACCUUGGUUCG
cluster_652_ext    GCCGUGAUCGUAUAGUGGUUAGUACUCUGUGCUGUGGCUGCAGCAACCUUGGUUCGAUAUACCCAAAUAAAA---
URS000067A424      GCCAUGAUCGUAUAGUGGUUAGUACUCUGCGCUGUGGCCGCAGCAACCUCGGUUCGAA---UCCGAGUCACGGCA
                   *** ************************* ******** ********** *******     ** * * *

cluster_677_frag                                          GAAACUGUUUAGACGGGCUCACAUCACCCCAUAAACA
cluster_677_ext    -------UUGCUUUUUUUUACAUAAGUAGACACAGGUGGGAAACUGUUUAGACGGGCUCACAUCACCCCAUAAACA
URS0000010A01      NNNNNUGUAGCUUACCUCCUCAAAGCAAUACAC-----UGAAAAUGUUUAGACGGGCUCACAUCACCCCAUAAACA
                          * ****   *   ** *   * ****      **** ********************************

cluster_703_frag      GUGAUCGUAUAGUGGUUAGUACUCUGCAUUGUG
cluster_703_ext    GCUGUGAUCGUAUAGUGGUUAGUACUCUGCAUUGUGA-------AACUGGGGUUCUUCAAUCAAACUUAAUGGGAAAAU
URS000044BAE3      GCCGUGAUCGUAUAGUGGUUAGUACUCUGCGUUGUGGCCGCAGCAACCUCGGUUC--GAAUCCGAGUCA-CGGCA----
                   ** *************************** *****        ***   *****   ****  * * *  ** *

cluster_782_frag                  GUAAUGGUUAGCACUCUGGUCUCUGAAUCCAG
cluster_782_ext    ----CUAGGGCAGUGGUAAUGGUUAGCACUCUGGUCUCUGAAUCCAGUAACUAGGGUGUCUCUCUUGAUAAAUCCC
URS0000415026      GGUUCCAU----GGUGUAAUGGUUAGCACUCUGGACUCUGAAUCCAGCGAUCCGAGUUCAAAUCUCGGUGGAACCU
                       * *     *  ******************* ************  *   * **     *** * *  * **

cluster_815_frag   CAGAGUGUAGCUUAACAUAAAGCACCCAACUUACACUUAGGAGAUUUCAACUCAACUUGACC
cluster_815_ext    CAGAGUGUAGCUUAACAUAAAGCACCCAACUUACACUUAGGAGAUUUCAACUCAACUUGACCACUCUGA
URS00001232F5      CAGAGUGUAGCUUAACAUAAAGCACCCAACUUACACUUAGGAGAUUUCAACUUAACUUGACCGCUCUGA
                   **************************************************** ********* ******

cluster_836_frag                 AUGGUCAGCACUCUGGACUCUGAAUCCAGCGAUCUGAGU
cluster_836_ext    UCGUGGAAGGGGUCAUGGUCAGCACUCUGGACUCUGAAUCCAGCGAUCUGAGUCACGGCACCAAGUUUC-AUG------
URS000064F320      GGCCCCAUGGUGUAAUGGUCAGCACUCUGGACUCUGAAUCCAGCGAUCCGAGUUC-------AAAUCUCGGUGGGACCC
                         * ** ** ********************************** ****         ** * **  **

cluster_883_frag   AGAAAUAUGUCUGAUAAAAGAGUUACUUUGAUAGAGUAAAUAAUAG
```

```
cluster_883_ext    AGAAAUAUGUCUGAUAAAAGAGUUACUUUGAUAGAGUAAAUAAUAGAGGUUUAUUCAGCAGACCUGAUA----
URS00003F05AD      AGAAAUAUGUCUGAUAAAAGAGUUACUYUGAUAGAGUAAAUAAUAGGAGCUUAAACCCC----CUUAUUUCUA
                   *************************** ******************  * ***  *  *    ** **
```

```
cluster_889_frag   CAGAGUGUAGCUUAACAUAAAGCACCCAACUUACACUUAGGAGAUUUCAACUCAACUUGACC
cluster_889_ext    CAGAGUGUAGCUUAACAUAAAGCACCCAACUUACACUUAGGAGAUUUCAACUCAACUUGACC-------
URS00001232F5      CAGAGUGUAGCUUAACAUAAAGCACCCAACUUACACUUAGGAGAUUUCAACUUAACUUGACCGCUCUGA
                   **************************************************** *********
```

```
cluster_907_frag          UGUUUAACCAAAACAUCAGAUUGUGAAUCUGACAACAGAGGGUU
cluster_907_ext    AAAUUCAUGUUUAACCAAAACAUCAGAUUGUGAAUCUGACAACAGAGGGUUUUCAUCCAUAAACAAAUG
URS00003361F9      UAAAUAUAGUUUAACCAAAACAUCAGAUUGUGAAUCUGACAACAGAGGCUUACGACCCCUUAUUUACCG
                    ** *   **************************************** **   * ** * *   *  *
```

```
cluster_985_frag   UCACUGUAAAGCUAAUUAGCAUUAACCUUUUAAGUUAAAGAUUGAG
cluster_985_ext    UCACUGUAAAGCUAAUUAGCAUUAACCUUUUAAGUUAAAGAUUGAGGAUU-CUAAACUCCCCUGCAGUAAA
URS0000172BA5      -CACUGUAAAGCUACUUAGCAUUAACCUUUUAAGUUAAAGAUUAAGAGAACCAACACCUCUUUACAGUGA-
                    ************* **************************** **     * * **  *  * **** *
```
